# Macrophage-TREM2 promotes cardiac repair by restricting the infiltration of CD8^+^ T cells via CXCL16-CXCR6 axis after myocardial infarction

**DOI:** 10.7150/thno.118014

**Published:** 2025-08-30

**Authors:** Linlin Zhang, Sheng Wang, Yu Ding, Tao Zheng, Jie Sheng, Yanshan Chen, Zhiyue Wang, Ximei Dai, Canbiao Wang, Long Ma, Jing Pan, Yunming Zhang, Longjiang Zhang

**Affiliations:** 1 Department of Radiology, Jinling Hospital, Affiliated Hospital of Medical School, Nanjing University, Nanjing, 210002, China.; 2 Department of Radiology, Jinling Hospital, Affiliated Hospital of Nanjing Medical University, Nanjing, 210002, China.; 3 Department of Radiology, Jinling Hospital, the First School of Clinical Medicine, Southern Medical University, Nanjing, 210002, China.; 4 Department of Radiology, Jinling Hospital, Affiliated Hospital of Nanjing University of Chinese Medicine, Nanjing, 210002, China.; 5 RealThinking Biotechnologies, Getang Avenue 11, Nanjing, 210002, China.

## Abstract

**Background:** Infiltrated CD8^+^ T cells following myocardial infarction (MI) are potential myocardial injury factors, triggering autoimmunity by binding to myocardial cell-specific proteins. It has been reported that autoimmunity after MI is restricted during the fibrosis period. Nevertheless, the relevant mechanisms of this process have scarcely been explored. Cell-cell communication analysis suggests that macrophages are the most promising group for restricting T cell activity. Unbiased single-cell sequencing data screening indicates that TREM2 is a leading gene significantly upregulated after MI. However, how TREM2 restricts the activity of CD8^+^ T cells remains unknown.

**Methods and Results:** The adoptive transfer of circulating CD8^+^ T cells after MI proved their autoimmune attribute of attacking cardiomyocytes. CD8 antagonistic antibodies and the anti-autoimmune drug Ozanimod effectively inhibited the infiltration of CD8^+^ T cells and significantly ameliorated the damage to cardiomyocytes after MI. In TREM2 KO mice, the infiltration of CD8^+^ T cells in the myocardium was significantly increased without influencing the number of Treg cells. The results of cell-cell communication revealed that CXCL16-CXCR6 was the most predominant receptor-ligand pair between macrophages and CD8^+^ T cells. The results of ELISA and Transwell indicated that TREM2 deficiency led to an increase in CXCL16 secretion, thereby enhancing the chemotaxis of CD8^+^ T cells. The KEGG analysis and Western Blot results demonstrated that the deficiency of TREM2 augmented the activation of the PI3K-AKT signaling pathway, thereby resulting in an increase in CXCL16 secretion. Moreover, TREM2 deficiency also reduced VEGFC secretion and cardiac lymphangiogenesis, thereby leading to the impairment of immune cell drainage. Additionally, TREM2 deficiency reversed Ozanimod's effect on inhibiting CD8^+^ T cell infiltration. The overexpression of TREM2 significantly decreased CD8^+^ T cell infiltration and improved cardiac function.

**Conclusions:** Macrophage TREM2 promotes cardiac repair by limiting the infiltration of CD8^+^ T cells and facilitating lymphangiogenesis.

## Introduction

Globally, the prevalence and mortality associated with heart failure (HF) consistently remain high 1. Myocardial infarction (MI), often serving as the primary trigger for HF, underscores the critical need for an enhanced understanding of the post-infarct cardiac repair mechanisms 1-3, which could pave the way for novel interventions to safeguard cardiac function.

The pivotal role of immunity in MI recovery has inspired researchers to explore immune-focused treatments, chiefly targeting excessive inflammation suppression or the stimulation of reparative processes subsequent to MI 4, 5. The inflammatory dynamic changes after MI are the result of the interplay between various innate and adaptive immune cells 6-8. Preclinical studies have showcased the potential benefits of Treg cell transfer, curbing autoinflammation driven by macrophages and T cells 8, 9, underscoring the significance of immunological synergy. Recent evidence suggests that adaptive immune cells such as T lymphocytes play a key role in the post-MI repair process. Among them, CD8^+^ T cells have an unfavorable impact on myocardial healing by binding to the myocardial cell-specific Myh6 protein, triggering autoimmunity and releasing granzyme B 10, 11. Macrophages, being central to the immune milieu post-MI, dominate both the inflammatory and proliferative phases 12, 13. Moreover, their phenotype closely mirrors the inflammatory state of the immune environment 6, 14. In view of the dual-edged nature of inflammation, it is of particular significance to actively explore the conserved functions of macrophages.

In the present study, we discovered that circulating CD8^+^ T cells after MI significantly inflicted damage on cardiomyocytes both *in vivo* and *in vitro*, verifying that CD8^+^ T cells trigger myocardial autoimmunity. Correspondingly, suppressing the infiltration of CD8^+^ T cells markedly mitigates the autoimmune assault. Macrophages are the most critical group in regulating T cell infiltration. We identified triggering receptor expressed on myeloid cells-2 (TREM2), a membrane-bound molecule found on macrophages 15, as a critical factor in taming post-MI autoimmune response. TREM2 drives macrophages to perform dual functions post-MI: inhibiting the intrusion of CD8^+^ T cells while catalyzing lymphangiogenesis. Throughout both the inflammatory and anti-inflammatory stages, TREM2 is prominently displayed on macrophage surfaces where it actively restrains the secretion of CXCL16, a crucial chemokine for the attraction of CD8^+^ T cells, leading to a lower levels of CD8^+^ T cells post-MI 16. Conversely, TREM2^+^ macrophages exhibit increased secretion of VEGFC, a potent inducer of lymphangiogenesis post-MI. This heightened VEGFC secretion stimulates the development of new lymphatic vessels, bolstering the cardiac drainage and facilitating inflammation resolution 10, 17. TREM2 also significantly reduced the Ly6C^hi^ monocytes/ Ly6C^lo^ macrophages ratio. Our investigation unveils fresh perspectives on the process of restricting autoimmunity following MI.

## Methods

### Animals

Wild type (WT) (Strain NO. N000013) and TREM2 knockout (KO) mice (Strain No. T003346) were purchased from GemPharmatech (Nanjing, China). Mice were housed in a pathogen-free environment, at a temperature of 20-26 °C, with humidity of 40%-60%, 12 h light/dark cycle, and were fed a standard laboratory diet. All animal studies described in this article were designed in accordance with the National Institutes of Health Guidelines for the Care and Use of Experimental Animals, which were approved by the Jinling Hospital Institutional Animal Care and Use Committee (DZYJKT202212230002).

### Mouse MI model

Minimal thoracotomy was employed to conduct myocardial infarction surgery in mice 18. Briefly, male or female mice were anesthetized with 2% isoflurane inhalation. Ketoprofen (5 mg/kg body weight) was administered intraperitoneally for analgesia. The sufficiency of anesthesia was assessed by respiratory rate and toe pinching. The limbs of the mice were immobilized and maintained in a supine position with the forechest exposed. The left front chest hair was removed and the skin was wiped with an alcohol cotton ball. An incision approximately 1 cm in length was made at the left side of the sternum and the edge of the pectoralis major muscle, and the surrounding skin was prepared for pocket suture. The skin was bluntly dilated, the muscles were separated, the intercostal muscles between the 3rd and 4th ribs were punctured, the ribs were expanded up and down, and the heart was rapidly extruded. The left coronary artery was permanently ligated using a 6-0 thread. Then the heart was immediately restored, the chest air was squeezed out, the sutures were tightened, and the chest was closed. The mouse was monitored until fully recovered. Mice in the experimental and control groups underwent surgery alternately. In each experiment, the order was random. After the surgery, the mice were placed in the same cage, and ear tags were used to distinguish the experimental and control groups. Euthanasia for all mice involved intraperitoneal injection of sodium pentobarbital at a dose of 200 mg/kg.

### Bone marrow transplantation (BMT)

Briefly, recipient mice (WT or TREM2 KO) received a total dose of 800 cGy of X-ray radiation. 5×10^6^ bone marrow cells from Donor mice were injected through the tail vein within 24 h. Recipient mice received antibiotics from 3 days before surgery to 1 week after surgery (neomycin at 1.0 mg/mL and polymyxin B at 100 μg/mL in drinking water). Recipient mice were allowed to recover for 8 weeks. The bone marrow transplantation mice were then subjected to MI surgery.

### Echocardiography

Two-dimensional and M-mode echocardiographic images were obtained in the parasternal long-axis view using a high-resolution *in vivo* Vevo3100 imaging system (FUJIFILM VisualSonics, Canada). The left ventricular inner diameters, including left ventricular (LV) ejection fraction (EF), LV LV end-diastolic volume, LV end-systolic volume, fractional shortening (FS), left ventricular end-diastolic diameter (LVEDD) and left ventricular end-systolic diameter (LVESD) were measured and calculated from three independent cardiac cycles.

### Mouse cardiac magnetic resonance imaging (MRI)

*In vivo* MRI studies were performed with a 9.4T MRI scanner (BioSpec 94/30 USR, Bruker, Germany). The ParaVision 360 acquisition workplace is delivered as part of the MR instrument. An 86 mm inner Transmit/Receive Volume Coil was used as the transmitter, and a Cardiac Array coil as a receiver for cardiac MRI resonance signal. ECG, respiration and anal temperature was measured by a basic life monitoring unit (SA Instruments, USA). The anal temperature of 37 °C was maintained by a body temperature conditioning system (ThermoFisher scientific, USA). Inhalational isoflurane/oxygen anesthesia (5% for induction and 1.0%-1.5% during MRI experiments) was delivered via a nose mask during the imaging procedure. Hearts were assessed by the T1 weighted imaging sequence of MRI (T1_FLASH sequence, time of repetition [TR]: 45ms; time of echo [TE]: 1.6 ms; averages:4; field of view [FOV]: 25 mm×25 mm; image size: 192×192, slice thickness: 1 mm). Movie sequences (cine_FLASH sequence, TR:5 ms; TE: 1.4 ms; Averages: 4; FOV: 25 mm ×25 mm; image size: 192×192, slice thickness: 1mm; movie frames: 15).

### Adenovirus construction and gene delivery *in vivo*

For the overexpression experiments, TREM2 KO mice were employed. The recombinant adenoviral vector harboring the TREM2 template plasmid (BC-4237, Brain Case), namely Ad-TREM2, and the control vector Ad-null were designed and synthesized by Brain Case (Shenzhen, China). The expression of the adenoviral vector was driven by the F4/80 promoter. Each TREM2 KO mouse was administered an intravenous injection via the tail vein with a dose of 1×10¹¹ viral genomes (vg) of adenovirus. MI was induced two weeks after the injection. The expression of TREM2 was evaluated through Western blot analysis and immunofluorescence staining on MI-7d.

### Flow cytometry analysis

Hearts were isolated on day 3 or day 7 post MI, and infarct and border areas were collected. The tissues were cut and cultured in medium containing 0.5 mg/mL collagenase A (10103586001, Roche, Switzerland), 0.5 mg/mL protease (Type XIV, P5147, Sigma, USA) and incubated at 37 °C for 20 min. The mixture was filtered using a 70 μm filter and centrifuged at 300 g for 10 min at 4 °C. Red blood cell lysate was used for a 5 min incubation and cells were obtained by centrifugation. Then the cells were re-suspended with 100 μL cell staining buffer and incubated with the corresponding antibody for 15-20 min in dark according to the manufacturer's instructions. Cells from each group were washed twice and analyzed in a flow cytometer at a constant flow rate and fixed collection time. Flow antibodies were purchased from Biolegend, anti-mouse CD45 (157213, 147711), anti-mouse/human CD11b (101228), anti-mouse CD3 (100203, 100205, 100235), anti-mouse CD19 (152409), anti-mouse CD64 (161006), anti-mouse CD206 (141705), anti-mouse Ly6C (128015), anti-mouse Ly6G (127607), anti-mouse CD11c (117309), anti-mouse CD4 (100411), anti-mouse FOXP3 (126403), anti-mouse CD8a (162310), anti-mouse TNF-α (506307), anti-mouse IFN-γ (505809), Rat IgG2a, λ Isotype Ctrl antibody (402305). The dosages of flow antibodies were precisely prepared in strict accordance with the dilution ratios recommended by the manufacturer.

### MHC tetramer staining

To validate whether α-myosin serves as an autoantigen that initiates autoimmunity following MI, we initially synthesized tetramers of six short peptides of α-myosin, namely MIYTYSGL, VIQYFASI, VQQVYYSI, AIMHYGNM, INFTNEKL, and VFFKAGLL 10, 19. The tetramer rapid switching assay was performed according to the manufacturer's instructions (QuickSwich^TM^ Quant H-2Kb Tetramer Kit, MBL, TS-7400-2F). Subsequently, heart samples from WT and TREM2 KO mice on MI-7d were collected. The infarcted and border regions were excised, digested, and dissociated into single-cell suspensions. The tetramers (APC-labeled) that had undergone the switching assay were co-incubated with the flow cytometry antibodies and the single-cell suspensions for 30 min. Subsequently, flow cytometry was employed to determine the proportion of CD8^+^ T cells that bound to the tetramers.

### Western blot analysis

Western blotting was performed as previously described with minor modifications 20. Cell or heart tissue extracts were separated on 12% SDS-PAGE and then transferred onto PVDF membranes. The PVDF membranes were incubated with a specific primary antibody overnight at 4 °C. The dilution factor for all primary antibodies was 1:1000. The following primary antibodies were used. Anti-TREM2 antibody (ab305103), anti-PI3 Kinase-gamma antibody (ab302958) and HRP anti-β actin antibody-loading control (ab20272) were bought from Abcam. Anti-phospho-PI3 Kinase p85 (Tyr458)/p55 (Tyr199) antibody (4228T), anti-phospho-AKT (ser473) antibody (4060T) and anti-Akt (pan) (C67E7) antibody (4691T) were bought from Cell Signaling Technology. VEGFC polyclonal antibody (PA5-29772) was bought from Thermo Fisher Scientific. The secondary antibody used was anti-rabbit IgG, HRP-linked antibody (7074S, Cell Signaling Technology). The secondary antibody dilution factor was 1:5000. ImageJ software was used to analyze the grayscale intensity of the immunoblot band. The band intensities were compared with β-actin.

### Histological analysis

Heart samples were fixed in 4% paraformaldehyde solution for 48 h. 6 μm paraffin sections were cut, dewaxing was performed before antibody staining. For immunofluorescent staining, sections were incubated with a specific primary antibody overnight at 4 °C. The following primary antibodies or kit were used. Anti-TREM2 antibody (ab305103, 1:100), anti-CD68 antibody (ab283654, 1:200), anti-Collagen I antibody (ab270993, 1:150), anti-α SMA antibody (ab7817, 1:150), anti-CD8a antibody (ab217344, 1:200), anti-CD3 antibody (ab135372, 1:300), anti-CD4 antibody (ab133616, 1:300), anti-Lyve1 antibody (ab218535, 1:200), anti-Ki67 antibody (ab16667, 1:200), anti-CD31 antibody (ab182982, 1:300) and anti-TNNI3 antibody (ab96684, 1:200) were bought from Abcam. TUNEL assay kit (C10617) was bought from Thermo Fisher Scientific. Slides were washed three times with PBS, and then stained with Goat anti-rabbit IgG H&L (Alexa Fluor 488) (ab150077), Goat anti-rabbit IgG H&L (Alexa Fluor 647) (ab150075) secondary antibodies for 1 h at room temperature. The dilution factor for the secondary antibodies was 1:1000. Slides were then stained with DAPI (Sigma-Aldrich, D9542) and Prolong Glass Antifade Mountant (P36984, Invitrogen). Images were acquired using laser confocal microscope (Leica, Germany).

We used Masson's trichrome staining to visualize fibrotic scars. A midline length-based method was used to determine the relative infarcted area 21. Analyses of infarct size, capillary density, lymphatic density, extent of early fibrosis, number of apoptotic cardiomyocytes and the count of CD8^+^ T cells were assessed using ImageJ software.

### Culture of bone marrow-derived macrophages (BMDMs)

Mouse BMDMs were isolated and differentiated into mature macrophages as previously described 22. Briefly, bone marrow cells were flushed out from mouse femurs and cultured in RPMI 1640 (Hyclone, MA, USA) supplemented with 10% fetal bovine serum (Hyclone), 100 μg/mL streptomycin, 100 U/mL of penicillin, and 10 ng/mL GM-CSF (HY-P7361, MedChemExpress, USA) in an incubator at a constant temperature of 37 °C, 5% CO_2_ and saturated humidity. For PI3K inhibition, BMDMs were treated with 5 μM PI3K/AKT-IN-1 (HY-144806, MedChemExpress, USA).

### Isolation of PBMCs from mice

Blood was collected from the eyes of mice and placed in an anticoagulant tube. PBMCs were isolated using the Mouse Peripheral Blood Lymphocyte Isolation Kit (C0029S, Beyotime). Briefly, the mouse blood was diluted with the diluent and gently added to the upper layer of the separation liquid. Centrifugation was performed at 450 g for 30 min at 20 °C. PBMCs were aspirated and washed twice with the washing solution.

### Magnetic bead sorting of CD8^+^ T cells

The PBMCs of sham and MI-7d mice were co-incubated with CD8 magnetic beads (130-117-044, Miltenyi Biotec). The MS column (130-042-201, Miltenyi Biotec) was placed on the MiniMACS^TM^ starting kit (130-090-312, Miltenyi Biotec) and moistened. The co-incubation liquid was added to the MS column and allowed to pass through slowly. The MS column was removed, rinsed with PBS, and collected. Centrifugation was carried out to acquire CD8^+^ T cells. They were either directly injected them into mice or resuspend them in the culture medium for 4 h of culturing. Each recipient mouse received an injection of 1×10^6^ CD8^+^ T cells. Seven days post-injection, the hearts of the mice were harvested for relevant assays.

### Determination of cell viability of mouse cardiomyocytes

Mouse cardiomyocytes were extracted as previous reports 23. Briefly, neonatal mouse hearts were dissociated using the Neonatal Heart Dissociation Kit (130-098-373, Miltenyi Biotec). The tissue was combined with the enzyme mixture and placed in a gentleMACS C tube and incubated at 37 °C for 15 min. The C tube was connected to the gentleMACS separator. After the procedure was terminated, the sample was resuspended in cell culture medium. It was filtered through a 70 μm filter. The cell suspension was centrifuged at 300 g for 5 min. Cardiomyocytes were isolated by gradient sedimentation. The cell suspension was inoculated in culture flasks for 90 min, and the non-adherent cardiomyocytes were collected and inoculated into 96-well plates and cultured for 24 h. The cultured CD8^+^ T cells were centrifuged, and the culture medium was collected. Half of it was used to resuspend the CD8^+^ T cells and added to the cardiomyocyte culture plates as the experimental group. The other half was directly added to the cardiomyocyte culture plates as the control group. After co-incubation for 24 h, all of the medium was replaced, and the cardiomyocytes activity was detected using the Cell Counting Kit-8 (CCK8, HY-K0301, MedChemExpress) and a microplate reader.

### Transwell migration assay

Spleens from WT mice were ground, filtered through a 40 μm filter, lysed to remove red blood cells, and centrifuged to obtain splenic T cells. The cells were re-suspended in 100 μL of medium and placed in a 5.0 μm polycarbonate membrane chamber (Corning Life Sciences, Corning, USA) for the following chemotaxis assay. Medium containing or not containing CXCL16 (HY-P7151, MedChemExpress, USA), WT or TREM2 KO BMDMs culture medium was added to the lower chamber. The plates were placed in the incubator for 24 h. The cells that migrated to the lower chamber were collected and the proportion of CD8^+^ T cells was analyzed by flow cytometry.

### siRNA transfection

24 h after BMDMs from TREM2 KO mice were seeded into 24-well plates, siRNA transfection was carried out. The transfection procedure was performed in accordance with the instructions of the Lipofectamine™ RNAiMAX transfection reagent (Cat. No. 13778150, Thermo Fisher Scientific). The final concentration of siRNA used for transfection was 10 nM. The BMDMs were allowed to internalize the siRNA in Opti-MEM for 24 h. Subsequently, the culture medium was replaced, and the BMDMs were cultured further. The siRNA sequence for CXCL16 was as follows: CAGGAGCACUGUCCUUAAA and UUUAAGGACAGUGCUCCUG. The transfection efficiency of the siRNA was evaluated by quantitative real-time polymerase chain reaction (qPCR).

### ELISA

Mouse IL-1β (AF-2040-A), IL-6 (AF-2163-A), TNF-α (AF-2132-A), TGF-β (AF-2686-A), and CXCL16 (AF2015-A) ELISA kits were used to analyze mice serum, mice heart tissue, cell culture medium samples, following the manufacturer's instructions (AiFang Biological, China).

### Bulk RNA sequencing (RNA-seq)

The groups obtained in RNA-seq consisted of two categories: the WT group and the TREM2 KO group. Each group contained three biological replicates, denoted as WT1, WT2, WT3 for the WT group and KO1, KO2, KO3 for the TREM2 KO group. MI-7d hearts were harvested from three male WT mice and three male TREM2 KO mice. Cardiac tissue digestion was performed according to the method of 'Flow Cytometry Analysis', and CD45^+^CD11b^+^CD64^+^ macrophages were obtained by flow sorting 24. Total RNA was extracted using TRIzol reagent (Invitrogen) according to the manufacturer' s protocol. RNA purity and quantification were evaluated using the NanoDrop 2000 spectrophotometer (Thermo Scientific, USA). RNA integrity was assessed using the Agilent 2100 Bioanalyzer (Agilent Technologies, Santa Clara, CA, USA). Then the libraries were constructed using VAHTS Universal V6 RNA-seq Library Prep Kit according to the manufacturer's instructions.

The libraries were sequenced on an Illumina Novaseq 6000 platform and 150 bp paired-end reads were generated. Raw reads in fastq format were first processed using fastp and the low-quality reads were removed to obtain the clean reads. The clean reads were mapped to the reference genome using HISAT2. FPKM of each gene was calculated and the read counts of each gene were obtained by HTSeq-count. PCA analysis was performed using R (v3.2.0) to evaluate the biological duplication of samples. Differential expression analysis was performed using the DESeq2. Moderated estimation of fold change and dispersion for RNA-seq data with DESeq2. Q value < 0.05 and foldchange > 2 or foldchange < 0.5 was set as the threshold for significantly differential expressed genes (DEGs).

### Single-cell RNA sequencing (scRNA-seq)

Sham surgery hearts and MI-3d, MI-7d, MI-14d hearts from male WT mice and MI-7d hearts from male TREM2 KO mice were collected. Cells other than cardiomyocytes were collected from the infarct region (infarct core and border zone). Then the cells were loaded into the 10× Genomics Chromium system for library construction. The Chromium Single Cell 3' Reagent Kit v2 (10× Genomics, USA) was used to generate single-cell gel beads in a simulation (GEM). Reverse transcription was performed in ProFlex PCR. A sequencing library was then constructed using cDNA. The constructed libraries were sequenced on the NovaSeq 6000 Illumina sequencing platform.

### Quality controlling, filtering and cluster analysis

Specifically, we filtered out cells that met any of the following criteria: < 200 or > 3000 unique genes detected or the average expression of mitochondrial genes > 25 reads. After removing of these outliers, 67263 cells were remained in sham and MI-3d, MI-7d, MI-14d datasets, 9534 cells remained in MI-7d TREM2 KO dataset, and 16680 cells remained in the 4 public datasets (GSM4307515, GSM4307530, GSM4307535, GSM4307540), respectively. Each dataset was normalized by “LogNormalize” using the Seurat R package. The Seurat standard comprehensive analysis method was used to remove the batch effects. First, we used "FindVariableFeatures" to select 2000 highly variable gene sets. The first 20 principal components were subsequently selected and used for integration. A combined dataset was created by finding the anchors between the individual datasets, which in turn creates a matrix of expressions for the batch correction.

Clustree was used to assess the stability of the clusters from a resolution of 0.2 to 1, and it was determined that a resolution of 0.8 gave the largest number of stable clusters with cells representing each donor in each cluster. “FindMarkers” was used to identify markers for each cluster. Cell types were then assigned to these clusters and annotated using these gene lists. The clustering results were visualized by using UMAP. Then we filtered doublets.

In-depth classification of T cell groups was performed according to previously reported methods 10. First, we clustered the cells using unsupervised clustering analysis, revealing their functional states. Then we grouped T cells by lineage. Finally, we identified Treg cells by marker gene FOXP3. Finally, we divided the T cell population into seven groups.

### Gene Ontology (GO) enrichment and Kyoto Encyclopedia of Genes and Genomes (KEGG) pathway enrichment analysis

The FindMarkers function was used to indentify differential genes (DEGs) for each cell subset by comparing the cell subset with other cells. A LogFC threshold of 2 DEGs were filtered, and p < 0.05 was used to retain DEGs were retained for scRNA-seq. A Log2 fold change > 2 or < -2 DEGs was applied for bulk RNA-seq. The ClusterProfiler R package was used to identify significantly enriched GO terms and KEGG pathways. P < 0.05 was set as the cutoff criterion for significant enrichment.

### Cell-cell communication analysis

The Cellchat package (v0.02) was used to analyze the cell-cell communications in macrophage and CD8^+^ T cell clusters. Cell-cell communication analysis was performed as previously described with minor modifications 25. "Secreted Signaling" was selected as the communication way. Gene expression data were projected onto protein-protein interaction (PPI) networks by identifying overexpressed ligand-receptor interactions. We focused on the upregulated ligand pairs in the TREM2 KO group. Permutation tests were performed to get probability values. The interactions of ligand-receptor pairs and the differences between WT and KO dataset were visualized in the form of a circle plot.

### Statistical analysis

The animal study design was based on published articles and our experience. In this study, mice were randomly allocated into experimental and control groups using simple randomization. GraphPad Prism 8.0 software was used for statistical analysis. All data are presented as the mean ± SEM. Data normality was determined by the Shapiro-Wilk test 26. For datasets that meet the criteria of a normal distribution, we performed the following tests. Unpaired Student t test was used for comparisons between two groups. For comparisons between multiple groups, analyses were performed using one-way analysis of variance (ANOVA) followed by Tukey 's multiple comparisons test. P < 0.05 was considered significant.

## Results

### Restricting the infiltration of CD8^+^ T cells mitigates autoimmune myocardial injury

**Figure 1:**
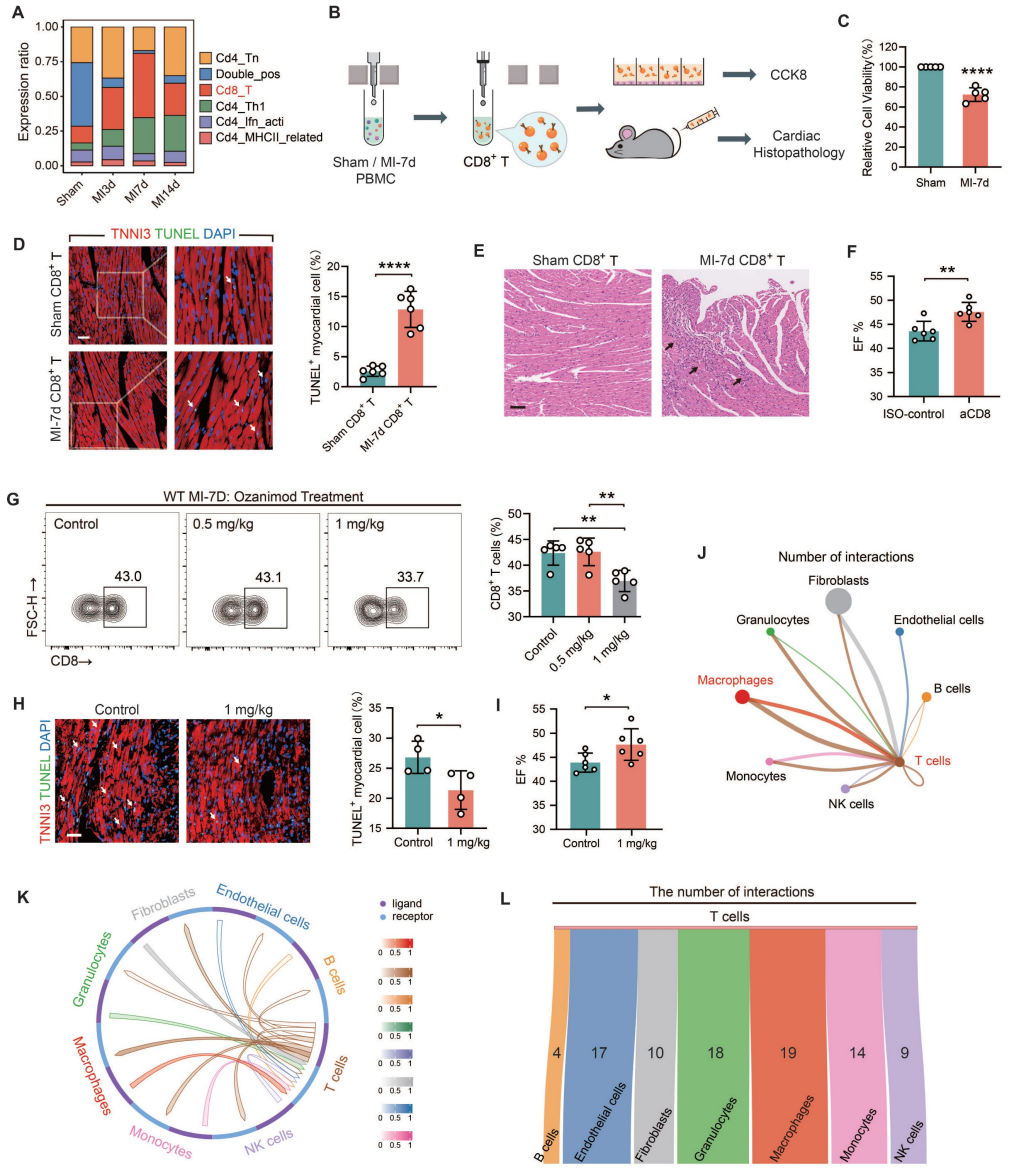
** Limiting the infiltration of CD8^+^ T cells mitigates autoimmune-induced myocardial injury after MI. (A)** The proportion of T cell subsets in single-cell sequencing (scRNA-seq) data of the four groups.** (B)** CD8^+^ T cells were isolated from the peripheral blood mononuclear cells (PBMC) of sham and MI-7d mice using magnetic beads, co-cultured with cardiomyocytes or injected into normal mice via the tail vein, and subsequent detection were performed.** (C)** The results of CCK8 indicated the viability of cardiomyocytes after 24 h with or without CD8^+^ T cells. Hearts of mice 7 days after adoptive transfer of CD8^+^ T cells: **(D)** The immunofluorescence staining results of apoptotic cardiomyocytes (n = 6, Scale bar: 50 μm); **(E)** HE staining of the hearts (Scale bar: 50 μm) under the effect of CD8 antagonistic antibody:** (G)** The proportion of CD8^+^ T cells in hearts after continuous intraperitoneal injection of Control, 0.5 mg/kg, and 1 mg/kg Ozanimod for 7 days after MI (n = 5). MI-7d hearts of the Control and 1 mg/kg Ozanimod groups: **(H)** Immunofluorescence staining of TNNI3 and TUNEL (n = 4, Scale bar: 50 μm);** (I)** EF% statistics (n = 6).** (J)** Cell-cell communication analysis was conducted using MI-7d scRNA-seq data, showing the number of interactions between each cell population and T cells.** (K)** CellCall results showed the ligand-receptor interactions between T cells and other cells.** (L)** The Sankey diagram illustrates the number of receptor - ligand pairs between each cell population and T cells. Data are presented as the mean ± SD. Student unpaired t tests or one-way ANOVA, Tukey test. *p < 0.05, **p < 0.01, ****p < 0.0001.

The single-cell sequencing data of mouse heart samples from sham, MI-3d, 7d, and 14d (*Figure S1*) were analyzed to identify the changing trends in the infiltration abundance of CD8^+^ T cells. The results showed that the proportion of CD8^+^ T cells significantly expanded after MI and peaked at MI-7d. Subsequently, the proportion started to decline (*Figure 1A*). To elucidate whether CD8^+^ T cells can initiate myocardial autoimmunity after MI, CD8^+^ T cells were obtained from the peripheral blood mononuclear cells of sham and MI-7d mice through magnetic bead sorting and were either co-cultured with mouse cardiomyocytes or intravenously injected into normal mice (*Figure 1B*). Flow cytometry analysis demonstrated, the successful isolation of CD8^+^ T cells using magnetic beads (*Figure S2A*). The *in vitro* experimental results suggested that, in contrast to the CD8^+^ T cells from sham mice, the presence of MI-7d CD8^+^ T cells significantly impaired the viability of cardiomyocytes (*Figure 1C*). In the myocardial slices of mice subjected to adoptive transfer of MI-7d CD8^+^ T cells, a large number of apoptotic cardiomyocytes, as well as inflammatory cell infiltration, distorted myocardial fiber arrangement, and fibrotic signs were observed. However, the myocardial slices of mice that received Sham CD8^+^ T cells presented no significant pathological changes (*Figure 1D-E and S2B*). The quantity of CD8^+^ T cell infiltrations in the hearts of the two groups showed no significant difference (*Figure S2C*). These findings indicate that the expanded CD8^+^ T cells after MI trigger an autoimmune response against cardiomyocytes, causing direct damage. After effectively eliminating CD8^+^ T cells using anti-CD8 antagonistic antibodies (*Figure S2D*), the cardiac function at MI-7d was significantly improved (*Figure 1F*); the number of apoptotic cardiomyocytes in the infarct border / non-infarct area was significantly reduced (*Figure S2E*); and the infarct area was significantly decreased (*Figure S2F*). Ozanimod is an anti-autoimmune drug approved by the FDA for the treatment of inflammatory bowel disease. It functions by sequestering peripheral lymphocytes and preventing their transport to inflammatory tissue sites 27. Regarding MI, we proved that 1 mg/kg of Ozanimod significantly reduced the infiltration of CD8^+^ T cells in the hearts of WT mice (*Figure 1G*), significantly decreased the number of apoptotic cardiomyocytes (*Figure 1H*), and significantly improved cardiac function at MI-7d (*Figure 1I*). This indicates that inhibiting the infiltration of CD8^+^ T cells can significantly alleviate myocardial injury after MI. To clarify how the infiltration of CD8^+^ T cells is restricted, a cell-cell communication analysis of WT MI-7d scRNA-seq dataset was conducted. The results revealed that the communication signals between macrophages and T cells were the most enriched (*Figure 1J*). CellCall was employed to identify the ligand-receptor interactions between T cells and other cells (*Figure 1K*). The Sankey diagram illustrates the number of receptor - ligand pairs between each cell population and T cells. Notably, the number of such pairs between macrophages and T cells is the highest (*Figure 1L*). Therefore, we consider macrophages as the most promising cell population capable of restricting T cell-related autoimmunity.

### TREM2 was identified as a prominent gene with an upregulated expression profile on macrophages after MI

**Figure 2:**
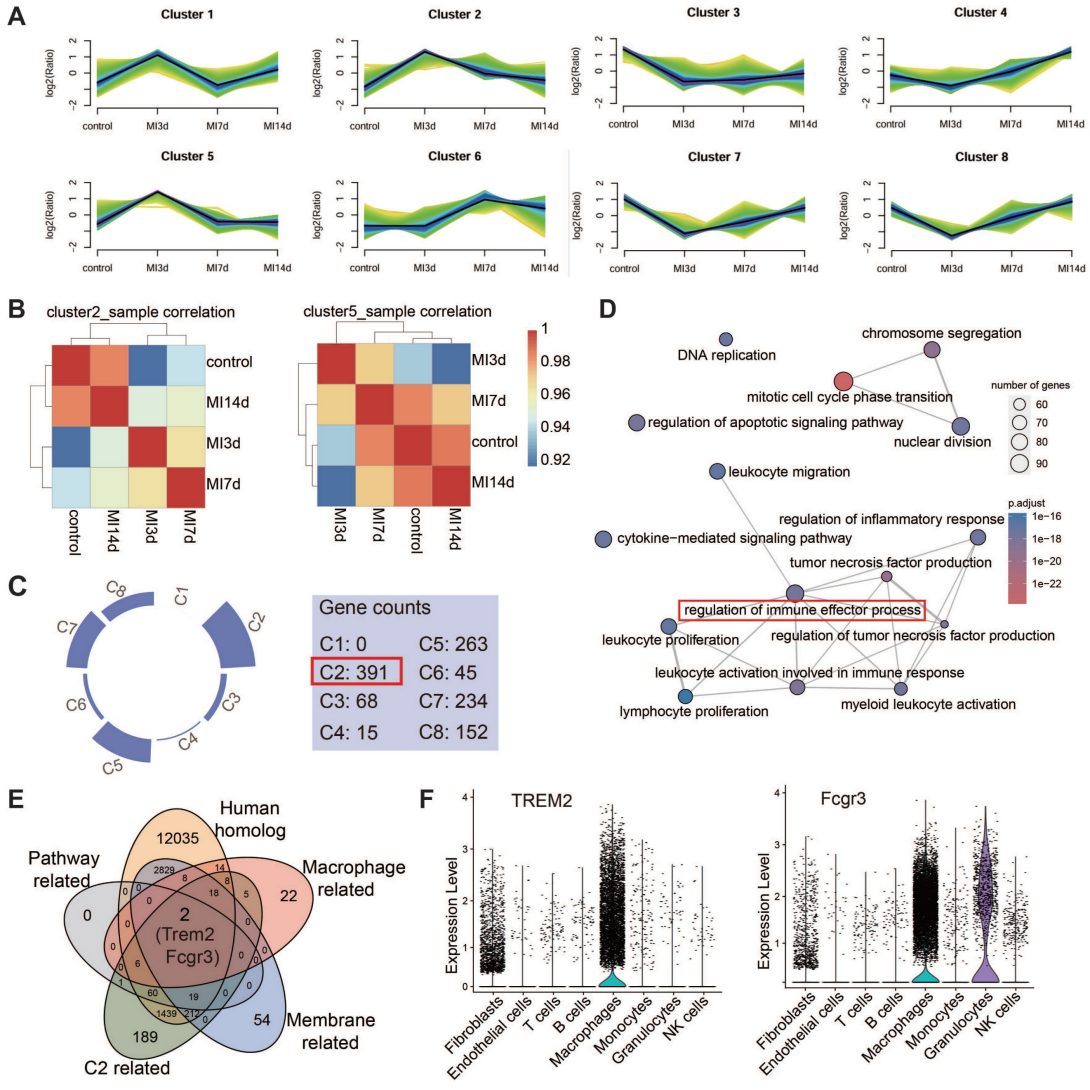
** Identification of TREM2 from single-cell RNA sequencing data. (A)** Eight gene sets of the four single-cell RNA sequencing datasets identified by Mfuzz analysis. **(B)** Clustering analysis results for Cluster2 (Left) and Cluster5 (Right) across the four samples. **(C)** Gene counts contained in each of the eight clusters. **(D)** Top 15 biological processes from GO enrichment analysis of Cluster2 genes. **(E)** Five criteria for gene selection. **(F)** Violin plots for TREM2 (Left) and FCGR3 (Right).

In light of the fact that the infiltration abundance of CD8^+^ T cells rose prior to MI-7d and dropped after MI-7d. Hence, we hope to screen fora factor expressed by macrophages that exhibit an initial increase and subsequent decrease in expression and is promising in restricting the infiltration of autoimmune CD8^+^ T cells. Four sc-RNAseq datasets (sham, MI-3d, MI-7d, MI-14d) of hearts from WT mice were incorporated into the analysis. The results of the Mfuzz analysis indicated that all genes could be grouped into eight gene clusters, with Clusters 2 and 5 exhibiting a pattern of initial increase followed by a decrease (*Figure 2A*). The results of the clustering analysis indicated that Cluster 2 demonstrated a comparatively weaker correlation with the sham and 14-day group (*Figure 2B*), suggesting that the dynamic expression of genes within Cluster 2 is more pronounced. Moreover, the number of genes that were expressed differentially in comparison with the control groups was found in this particular cluster (*Figure 2C*). Consequently, the gene selection focused on Cluster 2. Gene Ontology (GO) enrichment analysis pinpointed the top 15 biological processes associated with genes in Cluster 2, with "Regulation of immune effector process" occupying a central position (*Figure 2D*). Accordingly, an examination was conducted on homologous factors that are expressed in macrophages derived from humans and mice within this pathway and Cluster 2. This analysis focused on membrane proteins, which led to identification of TREM2 and FCGR3 (*Figure 2E*). TREM2 displayed the most distinctive pattern of expression in macrophages (*Figure 2F*). It has been documented that high levels of TREM2 within tumors are correlated with decreased infiltration of CD8^+^ T cells, but the specific association remains undefined 28. Hence, TREM2 was taken as the key research factor in this study. It has been claimed that TREM2 is related to the decreased infiltration of CD8^+^ T cells, yet the specific association remains ambiguous 28. Hence, TREM2 is taken as a research object in this study.

### TREM2 is highly expressed on cardiac macrophages after MI in both humans and mice

**Figure 3:**
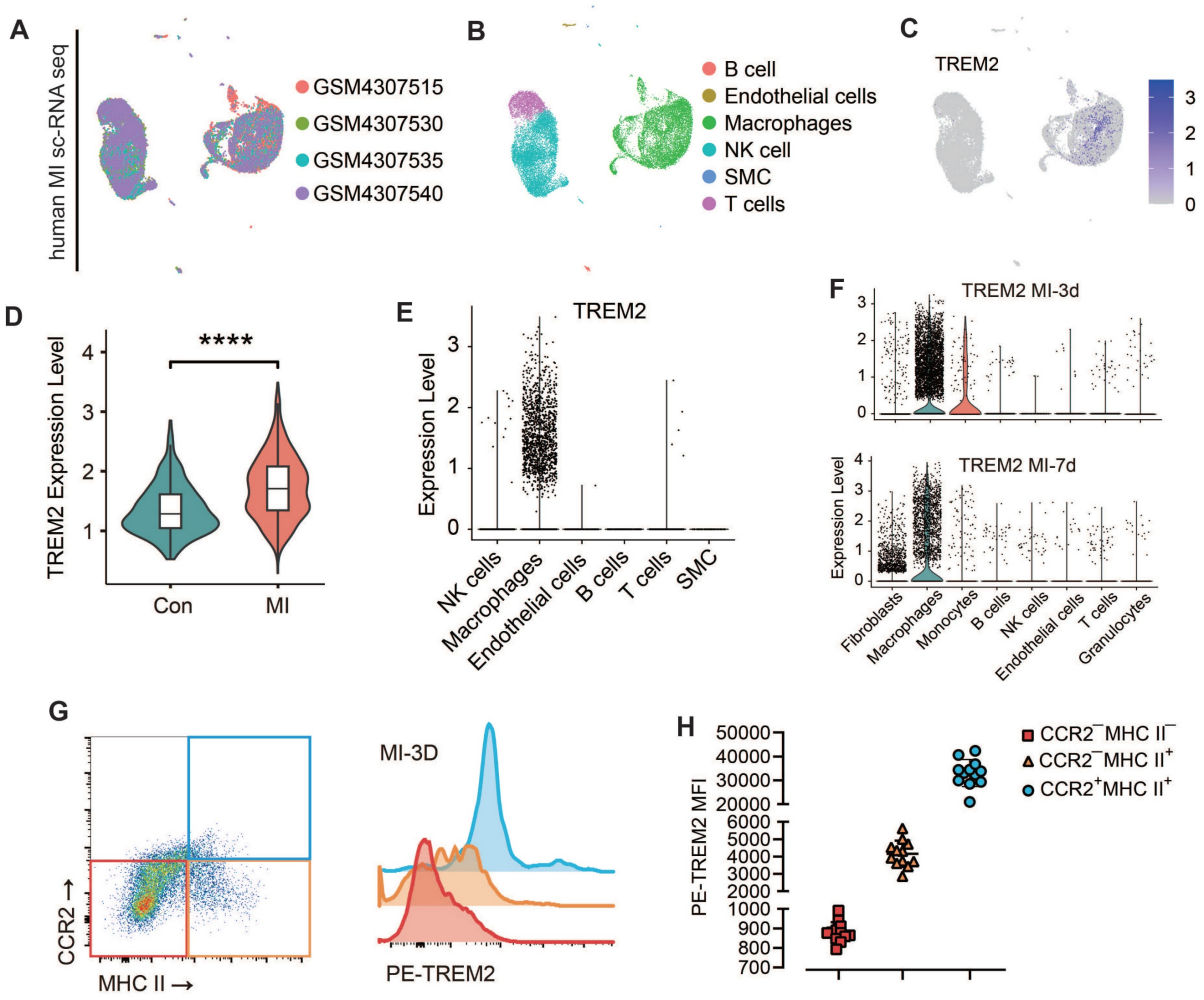
** TREM2 is highly expressed on cardiac macrophages after MI in both humans and mice.** Analysis of human infarcted heart scRNA-seq datasets from GSE145154: **(A)** UMAP plot and **(B)** cell clustering map. **(C)** TREM2 expression within macrophage cluster. **(D)** Quantified expression level of TREM2 comparing control (Con, GSM4307515) and MI (GSM4307530, GSM4307535, GSM4307540). **(E)** Violin plot of TREM2 expression across various cell populations (SMC, smooth muscle cells). **(F)** Violin plots of TREM2 expression across different cell populations in scRNA-seq data of MI-3d and MI-7d mice. **(G)** TREM2 expression in resident macrophages (CCR2^—^) and myeloid-derived macrophages (CCR2^+^) in the infarcted hearts. **(H)** TREM2 mean fluorescence intensity (MFI) in the three macrophage types (n = 12). Data are presented as the mean ± SD. One-way ANOVA, Dunnett's test **(H)** or Wilcoxon signed-rank test **(D)**. ****p < 0.0001.

The expression of TREM2 in humans post-MI was additionally validated using publicly accessible data from the GSE145154 database (*Figure 3A-C*). scRNA-seq data from four human hearts demonstrated that TREM2 expression was markedly enhanced post-MI in comparison to normal hearts (*Figure 3D*). This expression was observed to be almost exclusively confined to macrophages (*Figure 3E*).

This finding demonstrates that the upregulation of TREM2 expression in response to MI is a conserved phenomenon in human. For mice, western blot analysis revealed that, at day 3 and day 7 following MI, there was a pronounced upregulation of TREM2 expression of infarcted heart (*Figure S3A-B*). scRNA-seq analysis confirmed that TREM2 was predominantly expressed in macrophages (*Figure 3F*), and by day 3 following MI, macrophages had adopted a more pronounced pro-inflammatory profile compared to those at day 7 (*Figure S3C*). The findings of the immunofluorescence analysis provided additional support for the increased expression of TREM2 on macrophages at day 3 and 7 post-MI (*Figure S3D*). scRNA-seq from sham mice indicated that, under sham operation conditions, TREM2 was predominantly expressed by mononuclear macrophages (*Figure S3E*). Flow cytometry was employed to gain greater insight into the specific immune cell types expressing TREM2 in the infarcted heart. The findings revealed that the highest levels of TREM2 expression were observed in myeloid-derived cells (CD11b), particularly in macrophages (CD64). Additionally, low levels of TREM2 expression were detected in dendritic cells (CD11c). The expression of TREM2 was found to be minimal in T cells (CD3) and B cells (CD19) (*Figure S3F*). One more flow cytometry demonstrated TREM2 was highly expressed on myeloid-derived macrophages, rather than resident macrophages (*Figure 3G-H*).

### TREM2 deficiency enhances the infiltration of CD8^+^ T cells in heart tissue post-MI in male mice

**Figure 4:**
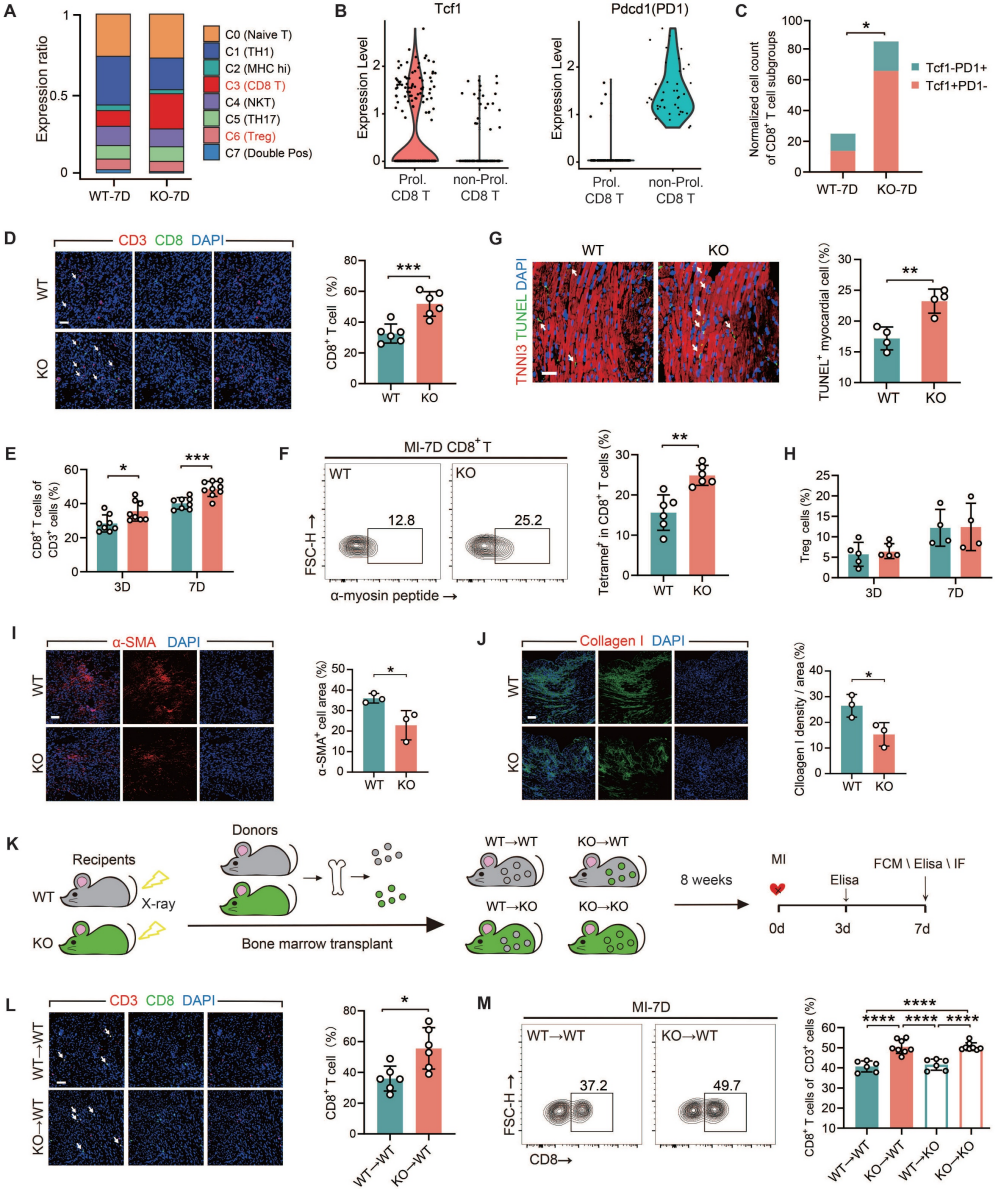
** TREM2 deficiency exacerbates CD8^+^ T cell infiltration in the infarcted area. (A)** T cell subset proportions of total T cells: scRNA-seq in WT / TREM2 KO mice, MI-7d. **(B)** Tcf1 and Pdcd1 gene expression profiles and **(C)** normalized counts of Tcf1^+^PD1^-^CD8^+^ / Tcf1^-^PD1^+^CD8^+^ T cells in scRNA-seq. **(D)** Immunofluorescence staining and quantification: CD8^+^ / CD3^+^ in infarcted hearts, MI-7d (Scale bar: 25 μm, n = 6). Flow cytometry analysis of **(E)** CD8^+^ T cells in WT / KO infarcted hearts at MI-3 / 7d (n = 8); **(F)** α-myosin peptide-tetramer binding CD8+ T cells from WT and TREM2 KO MI-7d hearts (n = 6). **(G)** Immunofluorescence images and quantification of TUNEL^+^ myocardial cells in infarct border zone, MI-7d (Scale bar: 50 μm, n = 4). Flow cytometry analysis of **(H)** Treg cells (CD4^+^FOXP3^+^) in WT / KO infarcted hearts at MI-3 / 7d (n = 5, 4). Immunofluorescence Images: **(I)**, α-SMA,** (J)**, Collagen I in infarcted and border zones, MI-7d (n = 3, Scale bar: 25 μm).** (K)** Diagram depicting bone marrow transplantation (BMT): WT/ KO mice (receivers) received lethal X-ray irradiation, then grafted with WT/KO BM cells. 8 weeks later, MI surgeries were conducted and relevant analysis was carried out on day 7. **(L)** Immunofluorescence staining and quantification: CD8^+^ / CD3^+^ in infarcted hearts, MI-7d (Scale bar: 25 μm, n = 6). Flow cytometry analysis: **(M)** CD8^+^ T cells (n = 6, 8) in BMT mice' infarcted heart at MI-7d (n = 6). WT→WT: Bone marrow cells from WT mice were transplanted into WT mice. WT→KO: Bone marrow cells from WT mice were transplanted into KO mice. KO→WT: Bone marrow cells from KO mice were transplanted into WT mice. KO→KO: Bone marrow cells from KO mice were transplanted into KO mice. Data are presented as the mean ± SD. χ^2^ test, Student unpaired t tests or one-way ANOVA, Tukey test. *p < 0.05, ***p < 0.001, ****p < 0.0001.

All of the experiments in this section were conducted using male mice only. We conducted a comparative analysis on two sets of the scRNA-seq data from WT and TREM2 KO mice at day 7 post-MI. We clustered the T cell populations into seven groups 29 and found that, following TREM2 deletion, a marked elevation noted in the relative abundance of T lymphocytes characterized by CD8 surface marker expression (*Figure 4A; Figure S4A*). The transcription factor TCF1 of CD8^+^ T cells is of crucial importance for maintaining antigen-specific immunity by providing dependable memory and expansion capabilities 30, 31. To ascertain the autoimmune attributes of CD8^+^ T cells in the MI heart with TREM2 deficiency, the expressions of TCF1 and the immune checkpoint protein PD1 were compared. The results demonstrated that the quantity of TCF1^+^PD1^-^CD8^+^ T (Prol. CD8 T) cells in the KO group was significantly higher than that in the WT group (*Figure 4B*). This indicates that the CD8^+^ T cell-related autoimmunity triggered by MI is enhanced after the deficiency of TREM2. The initial findings were validated by immunofluorescence staining and flow cytometry analysis of the infarcted hearts (*Figure 4D-E*). α-myosin is one of the cardiac autoantigens following MI 10, 19. On MI-7d, the number of CD8^+^ T cells that bound to peptides derived from α-myosin in the hearts of TREM2 KO mice was significantly greater than that in WT mice (*Figure 4F*). These findings suggest that after MI, CD8^+^ T cells initiate cardiac autoimmunity through binding to α-myosin. Furthermore, the absence of TREM2 leads to an exacerbation of this autoimmunity. Moreover, the quantity of TUNEL^+^ cardiomyocytes in the infarct border or non-infarct regions of KO mice was significantly augmented (*Figure 4G*). This indicates that the deficiency of TREM2 results in exacerbated myocardial damage. No discernible disparity emerged in TNF-α or IFN-γ secretion capabilities exhibited by CD8^+^ T lymphocytes sourced from TREM2 KO versus WT murine subjects, indicating comparable cytotoxic potencies between these CD8^+^ T cells (*Figure S4B-C*). It is noteworthy that the absence of TREM2 did not influence the discrepancies in the numbers of CD4^+^ T cells and Treg cells within the infarcted heart (*Figure S4D-F; Figure 4H*). The resolution of autoimmunity is accompanied by early fibrotic repair. Therefore, the expression of the fibroblast activation marker α-SMA and collagen I was evaluated at day 7 post-MI 6, revealing reduced early fibrotic repair following TREM2 deficiency (*Figure 4I-J*).

To elucidate the significance of myeloid immune cells expressing TREM2 in MI, we executed BMT between WT and TREM2 KO mice (*Figure 4K*). The absence of TREM2 was not associated with any impairment in the engraftment process. Eight weeks following the transplantation procedure, the mice began to display indications of coat color alteration. Subsequent protein blotting analyses of the transplanted mouse bone marrow substantiated the successful BMT engraftment (*Figure S4G-H*). The mice that had successfully undergone reconstruction were subjected to MI surgery. Analysis of MI BMT mice demonstrated that the selective ablation of TREM2 within the myeloid lineage resulted in enhanced infiltration by non-cytotoxic CD8^+^ T lymphocytes, while leaving the populations of CD4^+^ T cells and Treg cells largely unaffected in terms of their numerical counts (*Figure 4L-M; Figure S4I-L*). In mice with myeloid-specific TREM2 deficiency, early fibrotic repair was also found to be impaired (*Figure S4M-N*). The above-mentioned results suggest that TREM2 deficiency results in an enhanced infiltration of CD8^+^ T cells and attenuation of early fibrotic repair in infarcted hearts.

### TREM2-deficient macrophages promote CD8^+^ T Cells infiltration via CXCL16 secretion in male mice

**Figure 5:**
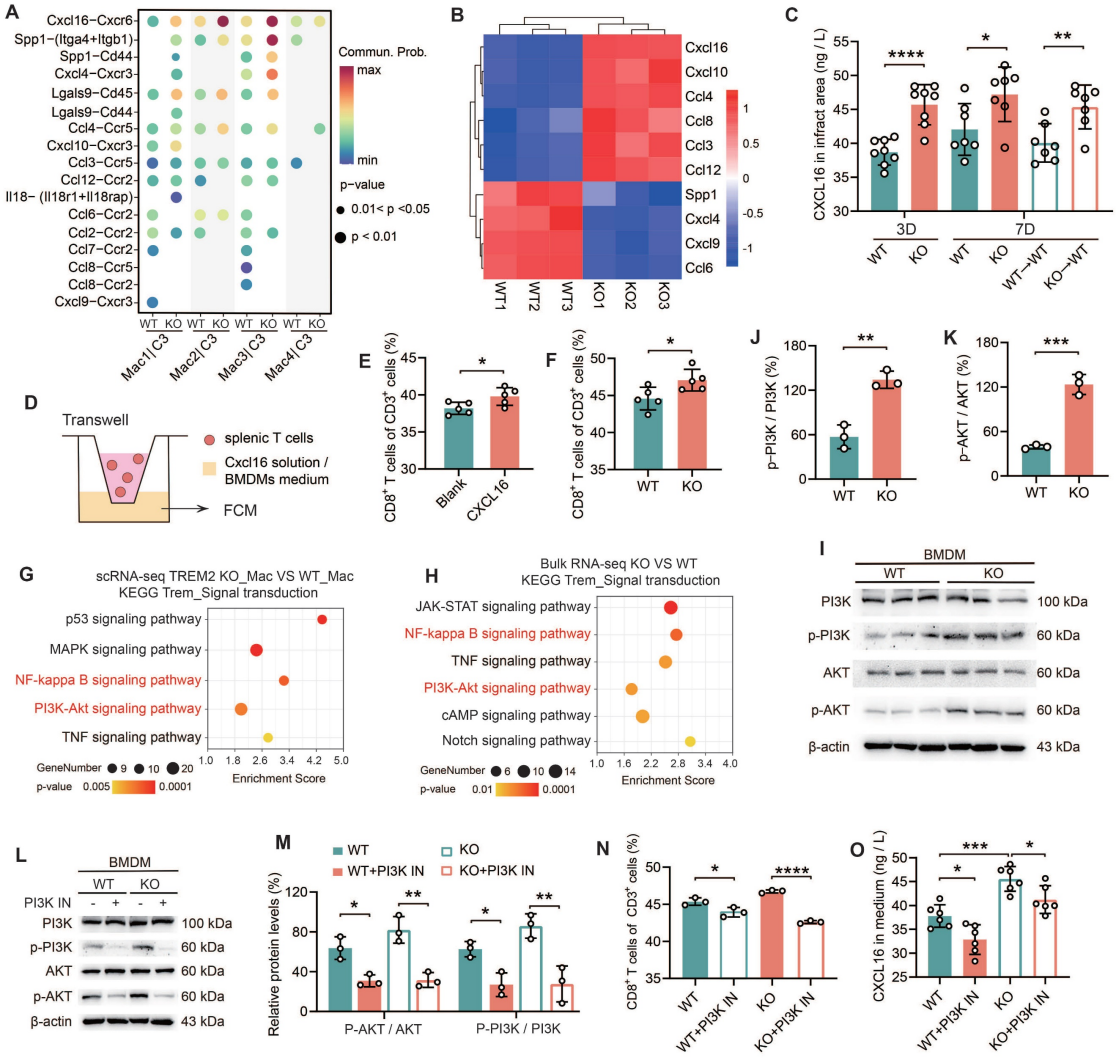
** Macrophages lacking TREM2 promote the infiltration of CD8^+^ T cells into the infarcted region via the secretion of CXCL16. (A)** Cell communication analysis: macrophage groups and CD8^+^ T cells in scRNA-seq, WT / TREM2 KO mice, MI-7d. **(B)** Heatmap: expression of ligands in **(A)**, bulk RNA-seq of macrophages (CD45^+^CD11b^+^CD64^+^) in WT/TREM2 KO hearts, MI-7d. **(C)** ELISA results of CXCL16 levels in infarct hearts of WT/KO/BMT Mice (n = 8, 7). **(D)** Schematic of the transwell experiment. The proportion of migrated CD8^+^ T cells in E) ± CXCL16 protein solution (n = 5), **(F)** BMDMs culture medium (n = 5). **(G)** In scRNA-seq, macrophages (Mac) were isolated from both the TREM2 KO group and the WT group. KEGG pathway analysis was subsequently conducted to compare the transcriptomic profiles of Mac from the two groups. **(H)** bulk RNA-seq KEGG analysis of the intracellular signaling pathways between TREM2 KO and WT data. I, **(L)** Western blot images of PI3K-AKT pathway-related proteins in BMDMs. Activation levels of **(J, K, M)** PI3K (n = 3) and AKT (n = 3). **(N)** Migration ratio of CD8⁺ T cells via Transwell (n = 3); (**O**) CXCL16 ELISA results in BMDMs culture medium ± PI3K inhibitor (PI3K IN) (n = 6). WT→WT: Bone marrow cells from WT mice were transplanted into WT mice. KO→WT: Bone marrow cells from KO mice were transplanted into WT mice. Data are presented as the mean ± SD. Unpaired student t test or one-way ANOVA, Tukey test. *p < 0.05, **p < 0.01, ***p < 0.001, ****p < 0.0001.

Tregs are immunosuppressive cells that play a crucial role in dampening inflammatory responses by curbing the proliferation and activation of CD8^+^ T cells 10, 32. However, TREM2 did not alter the quantity of Treg cells post-MI. The objective of this period was to ascertain whether there was a direct influence of TREM2 deficiency on the infiltration of CD8^+^ T cells. All experiments in this section were conducted using male mice only. Firstly, a ligand-receptor analysis was performed based on a large-scale interaction map 33. The ligands and receptors gene expression data derived from scRNA-seq were employed to categorize the interactions between macrophages and CD8^+^ T cells across distinct subpopulations (*Figure 5A; Figure S6A-B*). Notably, the CXCL16-CXCR6 ligand-receptor pair demonstrated the most substantial interaction discrepancies (*Figure 5A*). Moreover, CXCL16 and CXCR6 were both highly expressed in macrophages and T cell populations, respectively (*Figure S6C-D*). In order to gain further evidence, RNA-seq was conducted on macrophages (CD45^+^CD11b^+^CD64^+^) isolated from the hearts of KO and WT mice at day 7 post-MI. The data presented in Figure 5A yielded ten statistically significant ligands (*p* < 0.05), including CXCL16 (*Figure 5B*). It is noteworthy that CXCL16 expression was found to be upregulated in the KO group, in a manner that paralleled the trend observed in the ligand-receptor interaction (*Figure 5B*). Differential gene functional annotation from RNA-seq revealed strong enrichment of genes involved in T cell differentiation, T cell activation, and immune response, consistent with previous experimental results (*Figure S6E*). The elevated expression of genes associated with inflammation was observed in macrophages of the KO group (*Figure S6F*). These results suggest that TREM2-deficient macrophages promote the infiltration of CD8^+^ T cells into the infarct zone through the release of more CXCL16. ELISA analysis of the infarct hearts demonstrated that TREM2 deficiency was associated with sustained elevations in CXCL16 expression (*Figure 5C*). To verify the role of CXCL16 in the chemotaxis of CD8^+^ T cells, Transwell assays were performed (*Figure 5D*). The results demonstrated that the proportion of splenic CD8^+^ T cells migrating to the lower chamber was significantly higher in the presence of CXCL16 protein (*Figure 5E*). Furthermore, when culture supernatants from BMDMs from KO and WT mice were utilized, a stronger chemotactic effect was observed for the KO group on CD8^+^ T cells (*Figure 5F*).

Subsequently, BMDMs from TREM2 KO mice were transfected with either blank siRNA (si-nc) or CXCL16-targeting siRNA (si-CXCL16) (*Figure S6G*). The culture supernatants of these treated BMDMs were then utilized for Transwell assays. The results indicated that in the si-CXCL16 group, the percentage of CD8^+^ T cells that migrated to the lower chamber was significantly decreased (*Figure S6H*). These data suggest that bone marrow cells lacking expression of TREM2 exhibit enhanced secretion of CXCL16. To elucidate the mechanism by which TREM2 regulates CXCL16 expression, we employed KEGG analysis on both scRNA-seq and bulk RNA-seq data (*Figure 5G-H*). Upregulation was observed in signaling pathways including NF-κB, PI3K-AKT, and TNF signaling. The initial two pathways represent intracellular signaling cascades. It was observed that there was no significant difference in NF-κB p65 expression between WT and TREM2 KO BMDMs (*Figure S6I*). Subsequently, we conducted relevant experiments using a PI3K-AKT pathway inhibitor (PI3K IN). It was demonstrated that the PI3K-AKT pathway exhibited marked activation in TREM2 deficiency macrophages (*Figure 5I-K*), which aligns with a previous report 34. PI3K IN effectively inhibits the PI3K-AKT pathway activation (*Figure 5L-M*). Thereafter, we conducted Transwell assays utilizing the mentioned conditioned medium and proceeded to quantify CXCL16 protein levels. The results indicated that following the inhibition of the PI3K-AKT pathway, there was a notable reduction in CD8^+^ T cell migration, accompanied by a marked decline in CXCL16 expression (*Figure 5N-O*). The findings collectively illustrate that TREM2 deficiency macrophages secrete elevated levels of CXCL16 following PI3K-AKT pathway activation, which fosters CD8^+^ T cell migration into the infarct zone.

### TREM2-deficient macrophages inhibit lymphangiogenesis by reducing the secretion of VEGFC

**Figure 6:**
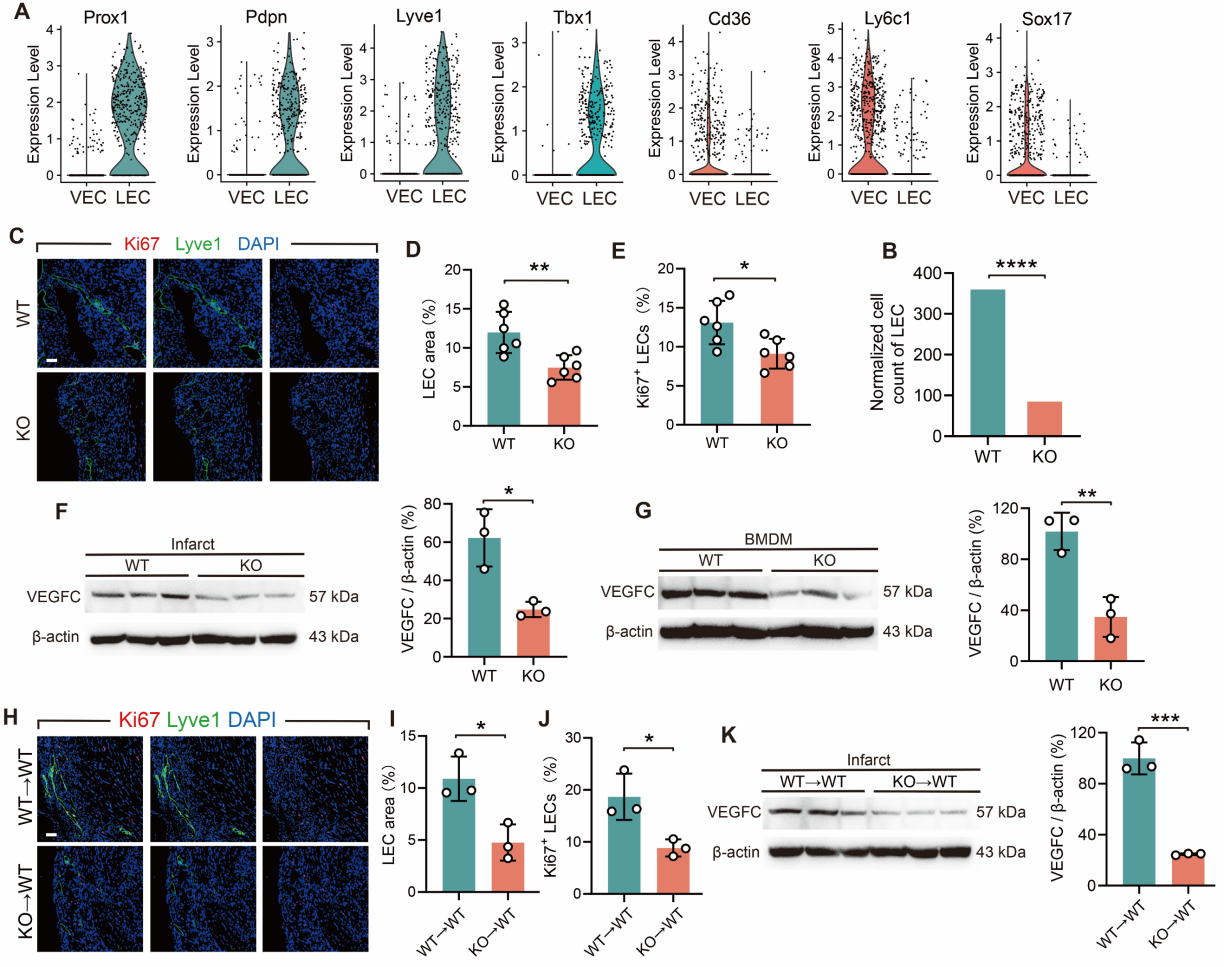
** TREM2 deficiency reduces lymphangiogenesis post-MI. (A)** Gene expression profiles of vascular endothelial cells (VECs) and lymphatic endothelial cells (LECs) in scRNA-seq. **(B)** Normalized cell counts of LECs. **(C)** Immunofluorescence staining images: Lyve1/Ki67 in infarct borders at MI-7d (Scale bar: 25 μm). Quantified** (D)** LEC area (Lyve1^+^ staining) and **E)** percentage of Ki67^+^ LECs (n = 6). Western blot imagines and quantitative analysis of VEGFC (WT vs. TREM2 KO) in **(F)** infarct hearts (MI-7d) and **(G)** BMDMs, (n = 3). **(H-J)** Lyve1 and Ki67 staining images and in infarct hearts at MI-7d of WT→WT and KO→WT mice quantitative analysis (Scale bar: 25 μm, n = 3). **(K)** Western blot imagines and quantitative analysis of VEGFC, infarct hearts, WT→WT and KO→WT mice, MI-7d (n = 3). WT→WT: Bone marrow cells from WT mice were transplanted into WT mice. KO→WT: Bone marrow cells from KO mice were transplanted into WT mice. Data are presented as the mean ± SD. χ^2^ test or unpaired student t test. *p < 0.05, **p < 0.01, ***p < 0.001, ****p < 0.0001.

The objective of the subsequent investigation was to ascertain whether an indirect interaction exists between TREM2^+^ macrophages and CD8^+^ T cells infiltration. The physiological functions of the cardiac lymphatic system encompass the reabsorption of extravasated fluid and the regulation of immune cell infiltration 35. Lymphangiogenesis provides a pathway for the exit of immune cells from the infarcted heart 2, 36. Therefore, we explored whether TREM2 deficiency affects lymphangio-genesis. In the analysis of MI-7d WT and TREM2 KO scRNA-seq, vascular endothelial cells (VECs) and lymphatic endothelial cells (LECs) were discriminated. The clustering approach was carried out according to the validated relevant marker genes (Figure S6J) 37, 38. For VECs, the marker genes include Cd36, Ly6c1, Sox17, etc. For LECs, the marker genes consist of Prox1, Pdpn, Lyve1, Tbx1, etc. (*Figure 6 A*). The result indicated that the quantity of LECs in TREM2 KO mice was significantly decreased compared to that in WT mice (*Figure 6 B*). Immunofluorescence staining demonstrated a marked decrease in the enumeration of lymphatic vessels in the infarct and border zones of TREM2 KO mice (*Figure 6C-E*). A key factor that promotes lymphangiogenesis is VEGFC, and macrophages are the main source of VEGFC secretion 39. We subsequently investigated whether TREM2 deficiency affects the expression levels of VEGFC. Western blot analysis revealed markedly attenuated expression intensities of VEGFC proteins within the infarcted hearts and BMDMs of TREM2-deficient mice compared to WT (*Figure 6F-G*). Additionally, the hearts of myeloid immune cells TREM2-deficient mice demonstrated reduced production of VEGFC and impaired lymphatic vessel formation (*Figure 6H-K*). The results of the study demonstrate that TREM2 plays a role in enhancing lymphangiogenesis by promoting VEGFC expression.

### TREM2 deficiency leads to deteriorated cardiac function after MI and counteracts the therapeutic effect of ozanimod in male mice

**Figure 7:**
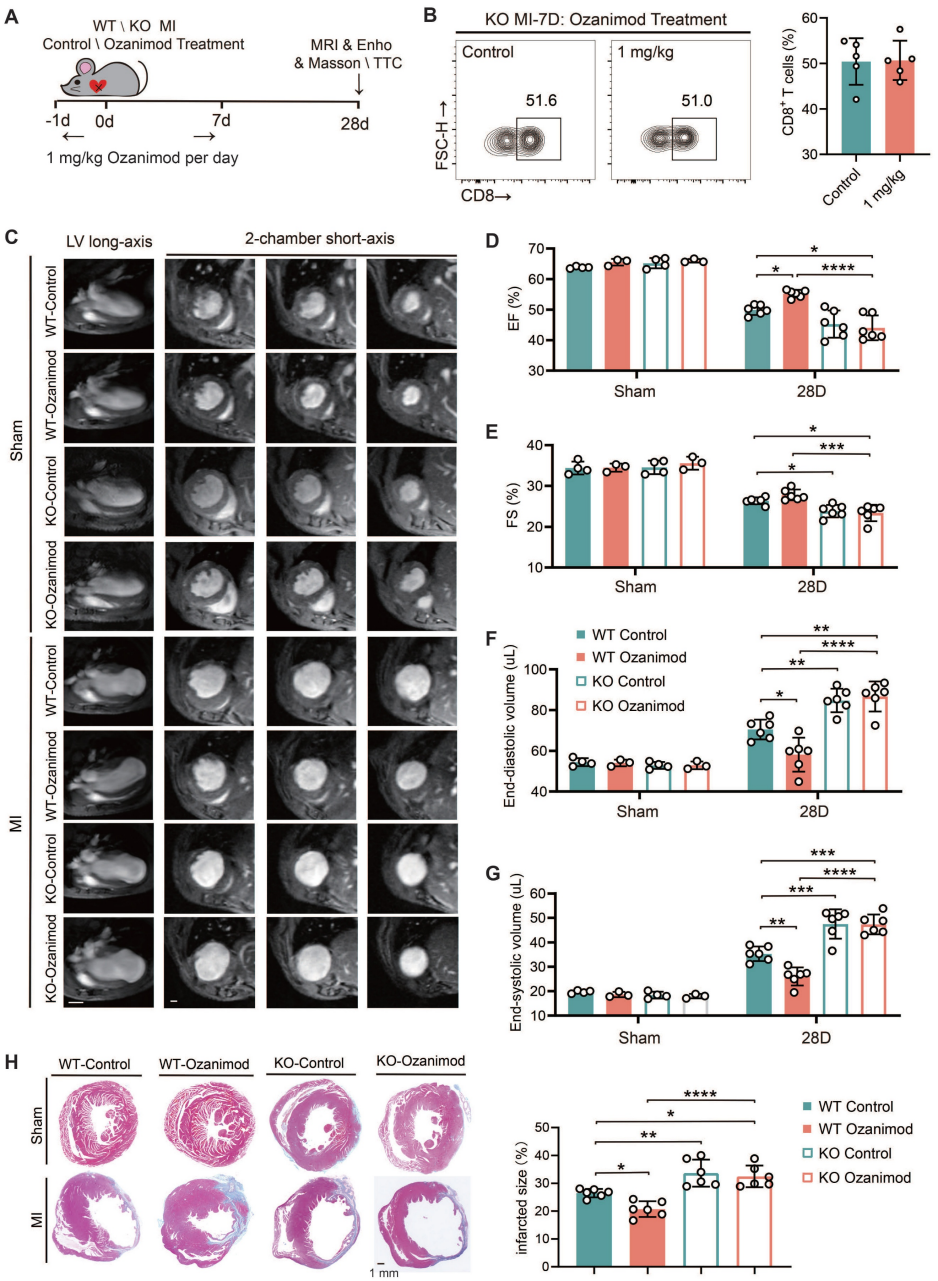
** TREM2 deficiency leads to worsened cardiac function and impaired repair after MI. (A)** Experimental design: WT and TREM2 KO mice were treated with or without 1 mg/kg of Ozanimod once daily from day -1 to day 7. Masson staining, magnetic resonance imaging (MRI), or echocardiography (Echo) were conducted at day 28. **(B)** Flow cytometry analysis of CD8^+^ T cells (n = 6, 8) in control and ozanimod treatment KO mice' infarcted hearts at MI-7d (n = 5). **(C)** Representative left ventricular (LV) long-axis and three consecutive 2-chamber short-axis end-diastolic MRI images of WT and KO mice. Scale bar: 3 mm (left), 0.5 mm (right). **(D)** Echocardiographic analysis of LV ejection fraction (EF), **(E)** LV fractional shortening (FS), **(F)** LV end-diastolic volume, **(G)** end-systolic volume (n = 4, 3, 6). **(H)** Masson staining on MI-28d and scar area quantification (n = 6, Scale bar: 1 mm). Data are presented as the mean ± SD. Student unpaired t tests. *p < 0.05, **p < 0.01, ****p < 0.0001.

All of the experiments in this section were conducted using male mice only. We have demonstrated that ozanimod can ameliorate the cardiac function and alleviate myocardial injury in WT mice after MI through inhibiting the infiltration of CD8^+^ T cells. However, TREM2 deficiency significantly promoted CD8^+^ T cell infiltration. In this section, we combined ozanimod treatment with WT and TREM2 KO mice respectively and compared their cardiac functions 28 days after MI. The experimental procedure for this section is illustrated in *Figure 7A*. The flow cytometry results of MI-7d indicated that ozanimod treatment failed to reduce the infiltration of CD8^+^ T cells in the hearts of TREM2 KO mice as it did in WT mice (*Figure 7B; Figure 1G*). Cardiac function assessments via 9.4T cardiac MRI and echocardiography were conducted. As demonstrated in the 9.4T cardiac MRI images, the ventricular wall of the infarcted group exhibited a considerable reduction in thickness, accompanied by a notable increase in the size of the cardiac cavity. Additionally, the contractility of the infarcted group was found to be severely compromised, in comparison to the sham group (*Figure 7C*). Furthermore, infarct size was greater and ventricular dilation was more pronounced in TREM2 KO mice compared to those of WT mice. Correspondingly, the echocardiographic results indicated that TREM2 deficiency had a more severe impairment on left ventricular function. Within the sham-operated cohort, no notable disparities emerged between WT and TREM2 KO mice concerning the examined parameters (*Figure 7D-G; Figure S7A-B*). Utilizing Masson's trichrome staining methodologies, it became evident that the extent of infarction in TREM2 KO mice markedly exceeded that observed in their WT counterparts (*Figure 7H*). Additionally, in WT mice, treatment with Ozanimod significantly decreased the infarct area and ameliorated the cardiac function. After Ozanimod treatment in TREM2 KO mice, no significant alterations were observed in cardiac function and infarct area (*Figure 7C-H*). This implies that the absence of TREM2 reversed the therapeutic effect of Ozanimod on MI.

### TREM2 deficiency increases the infiltration of CD8^+^ T cells in heart tissue post-MI and impairs cardiac function in female mice

In this part, we repeated the key findings described above using female mice to investigate whether there are significant sex differences in the effect of TREM2 on CD8^+^ T cells. In female TREM2 KO mice at MI-7d, the infiltration of CD8^+^ T cells was augmented, the number of apoptotic cardiomyocytes increased, the secretion of CXCL16 in myocardial tissue was elevated, and there was no significant difference in the infiltration of CD4^+^ T cells (*Figure S8A-D*). This suggests that gender differences have no significant influence on the regulation of CD8^+^ T cells by macrophage TREM2 after MI. The results of Masson's trichrome staining at MI-28d indicated that the infarct size in female mice with TREM2 deficiency was larger than that in WT mice (*Figure S8E*). The echocardiographic findings verified that deficiency of TREM2 led to impaired cardiac function in female mice (*Figure S8F-H*). The above results demonstrated that similar main research outcomes were acquired in both female and male mice.

### TREM2 overexpression improves tissue repair through restricting CD8^+^ T cells infiltration and lymphangiogenesis

**Figure 8:**
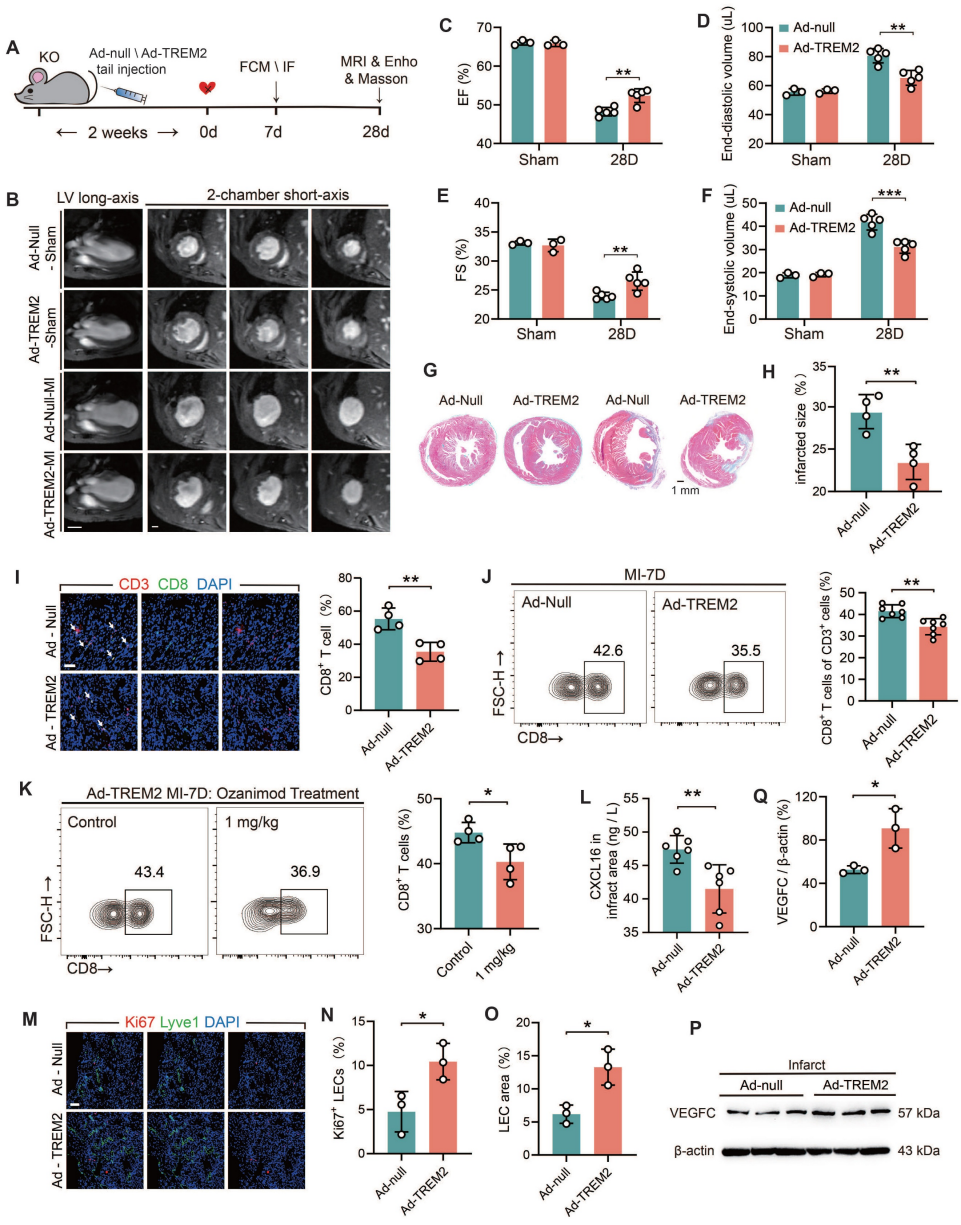
**TREM2 overexpression improves tissue repair through restricting autoimmunity. (A)** Experimental design: TREM2 KO MI mice were injected TREM2 adenovirus (Ad-TREM2) or control vector (Ad-null), followed by flow cytometry (FCM), immunofluorescence staining (IF), Masson staining, magnetic resonance imaging (MRI), and echocardiographic (Ehco) analysis at specified time points. **(B)** Representative 9.4 T cardiac MRI imagines in Sham / MI mice. Scale bar: 3 mm (left), 0.5 mm (right). **(C)** Echocardiographic analysis of left ventricular (LV) ejection fraction (EF), **(D)** LV end-diastolic volume,** (E)** fractional shortening (FS), **(F)** LV end-systolic volume (n = 3, 5). **(G)** Masson staining (Scale bar: 1 mm) and **(H)** quantification of infarct size, 28d post-MI (n = 4). **(I)** IF of CD3 / CD8 in the infarct border zone at MI-7d (n = 4, Scale bar: 25 μm).** (J)** FCM analysis showing the proportions of CD8^+^ T cells in infarct hearts, MI-7d (n = 7).** (K)** Flow cytometry analysis of CD8^+^ T cells in control and ozanimod treatment Ad-TREM2 mice' infarcted hearts at MI-7d (n = 4).** (L)** ELISA results of CXCL16, infarct areas, MI-7d (n = 6). **(M)** IF of Lyve1 and Ki67 in the infarct border zone (Scale bar: 25 μm). Quantified **(N)** percentage of Ki67^+^ lymphatic endothelial cells (LECs) and **(O)** LEC area (Lyve1 staining) (n=3). **(P)** Western blot images and **(Q)** quantification adjacent of VEGFC in the infarct hearts, MI-7d (n = 3). Data are presented as the mean ± SD. Unpaired student t test. *p < 0.05, **p < 0.01.

To further elucidate the beneficial role of TREM2 in the cardiac remodeling process, adenoviral vectors (Ad-null and Ad-TREM2) were administered via tail vein injection, and MI surgery was conducted two weeks later (*Figure 8A*). Western blot and immunofluorescence staining results confirmed the effective transfection of TREM2, which was predominantly expressed in macrophages within the infarcted heart (*Figure S9A-B*). The cardiac function of Ad-TREM2 mice was significantly improved compared to controls, while echocardiograms performed on the sham-operated cohort did not reveal any appreciable deviations from baseline readings (*Figure 8C-F; Figure S9C-D*). The application of 9.4T cardiac MRI and Masson's staining revealed a reduction in infarct size in the group exhibiting overexpression of TREM2 (*Figure 8B, G-H*). Furthermore, both flow cytometry and immunofluorescence staining supported that CD8^+^ T cell infiltration was reduced in the infarcted hearts following TREM2 overexpression (*Figure 8I-J*). This indicates that the expression of TREM2 contributes to restricting the infiltration of CD8^+^ T cells within the infarcted heart. Following TREM2 overexpression, KO mice with MI could be treated with Ozanimod, and the infiltration of CD8+ T cells in the heart was decreased (*Figure 8K*). And TREM2 overexpression reduced CXCL16 expression in the infarcted heart (*Figure 8L*). Additionally, enhanced lymphatic vessel formation and elevated VEGFC expression were indentified (*Figure 8M-Q*). These findings suggest that TREM2 promotes lymphangiogenesis by upregulating VEGFC expression, thereby providing a pathway for CD8^+^ T cell egress and additionally, to reduce its chemotactic effect on CD8^+^ T cells. Collectively, these dual functionalities serve to restrict CD8^+^ T cell infiltration in the infarct heart, thereby facilitating the restriction of autoimmunity and subsequent cardiac repair.

### TREM2 deficiency delays inflammation resolution after MI

The objective of this part is to ascertain the function of TREM2 in the immune microenvironment after MI. The concentrations of inflammatory cytokines in the serum of mice post-MI were quantified. The data demonstrated that expression levels of the pro-inflammatory cytokines IL-1β and IL-6 were elevated in TREM2-deficient mice at both day 3 and day 7. However, no significant change was observed in TNF-α levels, while the concentration of the repair factor TGF-β was reduced at day 7 (*Figure S10A-D*). We proceeded to evaluate the percentages of immune cell populations in the infarct hearts at day 3 and day 7 through the utilization of flow cytometry (*Figure S10E-H*). Ly6C^hi^ monocytes are cells with an inflammatory phenotype. They can initiate a strong inflammatory response and result in pathological remodeling. Ly6C^lo^ macrophages are cells with an anti-inflammatory phenotype, which release VEGF and TGF-β to facilitate repair processes 40. The results revealed a striking augmentation in the population density of Ly6C^hi^ inflammatory monocytes at day 7 in TREM2-deficient hearts, while the proportion of Ly6C^lo^ anti-inflammatory macrophages decreased (*Figure S5E-F*). There were no significant differences in neutrophils or CD206^+^ macrophages infiltrations over time (*Figure S9G;Figure S11A*). TREM2 deficiency resulted in a persistent increase inT cells infiltration (*Figure S10H*). These findings suggest that the transition from the inflammatory stage to resolution is delayed in the absence of TREM2, and cardiac autoimmunity may be exacerbated. At 7 days post MI, no significant differences were observed with regard to angiogenesis, as determined by CD31 (*Figure S11C*).

In mice exhibiting myeloid-specific deficiency in TREM2 (BMT mice), elevated levels of circulating pro-inflammatory cytokines were demonstrated following MI, while the level of the repair factor TGF-β exhibited a notable reduction at day 7 (*Figure S10I-L*). The results of flow cytometry demonstrated that the infarct area was characterized by enhanced autoinflammation. This was evidenced by an influx of inflammatory cells and T cells. Conversely, mice with resident cell-specific TREM2 deficiency exhibited reduced autoinflammation in the infarct zone, which was similar to the results observed in myeloid cells expressing TREM2 mice (*Figure S10M-N*). These findings reinforce the importance of TREM2, especially in myeloid macrophages, in mediating the resolution of autoinflammation and facilitating early fibrotic repair in MI tissue.

## Discussion

CD8^+^ T cells are not conducive to MI repair and aggravate myocardial injury by releasing granzyme B 41. Recent research indicated that after MI, CD8^+^ T cells possess the ability to attack the heart autoimmunologically, as they have an enhanced capacity to bind the myocardial cell-specific protein α-myosin 10. Under such circumstances, α-myosin is recognized as an autoantigen by CD8^+^ T cells, thereby triggering autoimmunity 19.

In this study, we proved that the increased level of CD8^+^ T cells triggered by MI significantly caused myocardial cells injury both *in vivo* and *in vitro*. Specifically manifested as reduced myocardial cell activity and the induction of myocarditis in mice. The MHC tetramer staining experiment verified that the binding of CD8⁺ T cells to α-myosin after MI initiated an autoimmune response, which significantly impaired cardiac function and repair. Correspondingly, inhibiting the migration of circulating CD8^+^ T cells to the myocardium significantly ameliorated this self-attack. Notably, the infiltration of CD8^+^ T cells after MI is restricted during the period from 7 to 14 days. Intercellular interactions determine the functional immune responses under homeostasis and inflammation 16. By means of scRNA-seq analysis, we discovered that macrophages were the principal cell population regulating T cell infiltration. Therefore, we conducted a screening process to identify potential factors in macrophages and identified TREM2. A previous study has indicated a certain correlation between TREM2 and the infiltration of CD8^+^ T cells, but the specific mechanism remains undefined 28. Our findings indicate that macrophage TREM2 consistently and stably exerts anti-CD8^+^ T cell effects. Upregulation of TREM2 endows macrophages with enhanced capabilities to restrain adaptive immunity and promotes lymphangiogenesis. Our findings also indicate that sustained targeting of macrophages following MI could exert stabilizing regulatory effects on tissue repair.

TREM2^+^ myeloid macrophages restrict CD8^+^ T cell infiltration into the myocardium, by reducing the production of CXCL16, thereby establishing an immunosuppressive microenvironment to facilitate post-MI repair. We unbiasedly identified the CXCL16-CXCR6 interaction between macrophages and CD8^+^ T cells in the infarcted hearts. Macrophage-TREM2 deficiency produced more CXCL16, which attracted more CD8^+^ T cells. Furthermore, we observed that TREM2^+^ macrophages inhibited CXCL16 expression by limiting the activation of the PI3K-AKT pathway. To the best of our collective expertise and literature review, this is the first report on the regulation of CXCL16 expression by TREM2. In terms of their functional characteristics, the chemotactic CD8^+^ T cells were mainly characterized by a TCF1^+^PD-1^—^ phenotype (stem cell-like CD8^+^ T cells, with dependable memory and expansion capabilities) and exhibited no significant cytotoxicity. This finding is in accordance with a previous report which suggested that this cell population was characterized by proliferation and autoimmunity, rather than cytotoxicity 10. Interestingly, we found that TREM2 did not affect the infiltration of Treg cells. Treg cells have potent immunosuppressive capabilities that can restrict the expansion and infiltration of CD8^+^ T cells 9. This observation underscores the remarkable specificity and conservation manifested in the CXCL16-CXCR6 pathway for the recruitment of CD8^+^ T cells. The data indicate that the limitation of CD8^+^ T cell recruitment mediated by TREM2^+^ macrophages plays a crucial role in establishing an immunosuppressive microenvironment establishment after MI. These macrophages are responsive to pro-inflammatory stimuli and furnish signals to restrict CD8+ T cell recruitment, indicating that they display characteristics similar to those observed in tumor-associated macrophages. This suggests that they may also participate in tumor-related immune suppression.

VEGF is a critical factor in the repair process following MI 39, 42. An increase in reparative factors indicates that the infarcted area is in the process of repair and healing. The exogenous administration of VEGFC and endogenous VEGFC secreted by myeloid macrophages have been demonstrated to enhance lymphangiogenesis after MI, thereby improving drainage function within the myocardium 39, 43. This helps alleviate myocardial edema and reduce the infiltration of inflammatory immune cells. 10, 44. These findings lend support to the notion that the reconstruction of a functional lymphatic network within the infarcted region is of pivotal importance for the repair process following MI 35. The present study demonstrated that TREM2^+^ macrophages facilitate the secretion of VEGFC, thereby promoting lymphatic vessel formation. These results contribute to the enhanced clearance and drainage of inflammatory immune cells, thus facilitating anti-inflammatory repair. To our knowledge, this is the first report on the influence of TREM2 on VEGFC and lymphangiogenesis. It has been previously demonstrated that TREM2^+^ macrophages facilitate the process of efferocytosis following MI 45, and this process can serve as an important reprogramming signal that induces macrophages to produce VEGFC 39. This provides substantial evidence for TREM2's role in promoting VEGFC expression.

Ischemic death of cells resulting from MI releases cardiac antigens, thereby triggering an autoimmune response and causing the heart to be attacked 46. The persistent anti-cardiac autoimmunity plays a crucial role in facilitating the progression to heart failure after MI 47. CD8^+^ T cells are regarded as one of the major players in autoimmunity 19. A considerable amount of researches support the pathological function of CD8^+^ T cells in post-MI repair 10, 41. Our study illustrates that macrophage TREM2 decreases the recruitment of CD8^+^ T cells by diminishing the secretion of CXCL16 and facilitating lymphangiogenesis, while the recruitment of various inflammatory immune cells is not significantly affected in the initial phase 10. TREM2 deficiency could reverse the inhibitory effect of the anti-autoimmune drug Ozanimod on the recruitment of CD8^+^ T cells. The anti-autoimmune effect exerted by TREM2 is persistent and spans both the inflammatory and anti-inflammatory phases. This functional property of TREM2 determines its compliance with the intrinsic mechanisms of post-MI repair, which renders TREM2 an attractive candidate for targeting therapeutic application. It is encouraging to note that human safety of the TREM2 agonist AL002C has been established 48, and it is advancing through Phase III of clinical evaluation for Alzheimer's disease. Further research is required to determine whether a TREM2 agonist has therapeutic effects in MI patients.

## Supplementary Material

Supplementary figures.

## Abbreviations

BMDM: bone marrow-derived macrophage
EF: ejection fraction
FS: fractional shortening
GO: gene ontology
HF: heart failure
KEGG: kyoto encyclopedia of genes and genomes
KO: knockout
LEC: lymphatic endothelial cells
LV: left ventricular
LVEDD: left ventricular end-diastolic diameter
LVESD: left ventricular end-systolic diameter
MI: myocardial infarction
PI3K IN: PI3K-AKT pathway inhibitor
scRNA-seq: single-cell RNA sequencing
si-CXCL16: CXCL16-targeting siRNA
si-nc: blank siRNA
TREM2: triggering receptor expressed on myeloid cells-2
VEC: vascular endothelial cells
WT: wild type
