# Supplementary material for: Macrophage-TREM2 promotes cardiac repair by restricting the infiltration of CD8+ T cells via CXCL16-CXCR6 axis after myocardial infarction
Source: Theranostics. 2025 Aug 30;15(18):9580–600. doi: 10.7150/thno.118014 (PMC12486271; doi:10.7150/thno.118014)
Supplement: Supplementary file 1 — Supplementary figures. [file thnov15p9580s1.pdf]

Supplementary Materials

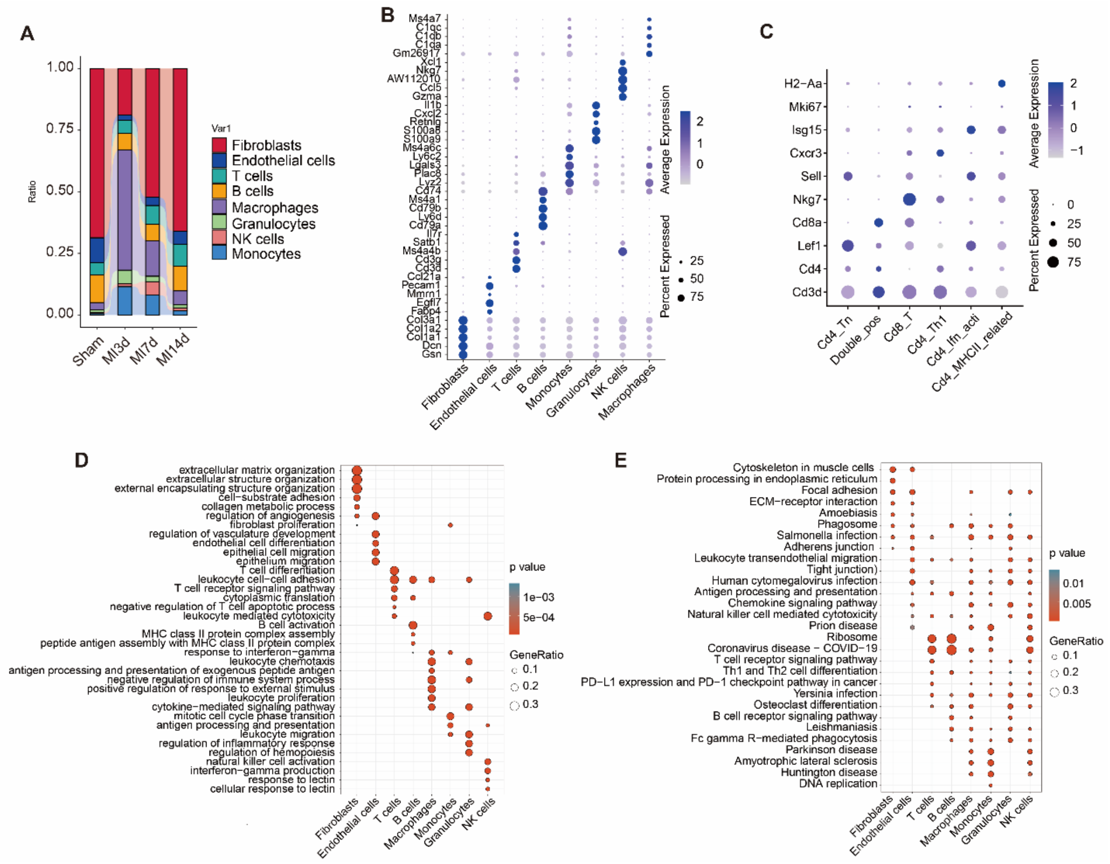

Figure S1 Basis for single-cell sequencing cell annotation. A) Fraction of each immune cell population relative to all cells during MI progression. **B)** Dot plot showing distinct gene expression signatures for each cell type. **C)** Dot plot showing distinct gene expression signatures for each T cell type. **D)** GO (biological processes) and **E)** KEGG analysis results for the top 50 genes of each cell type.

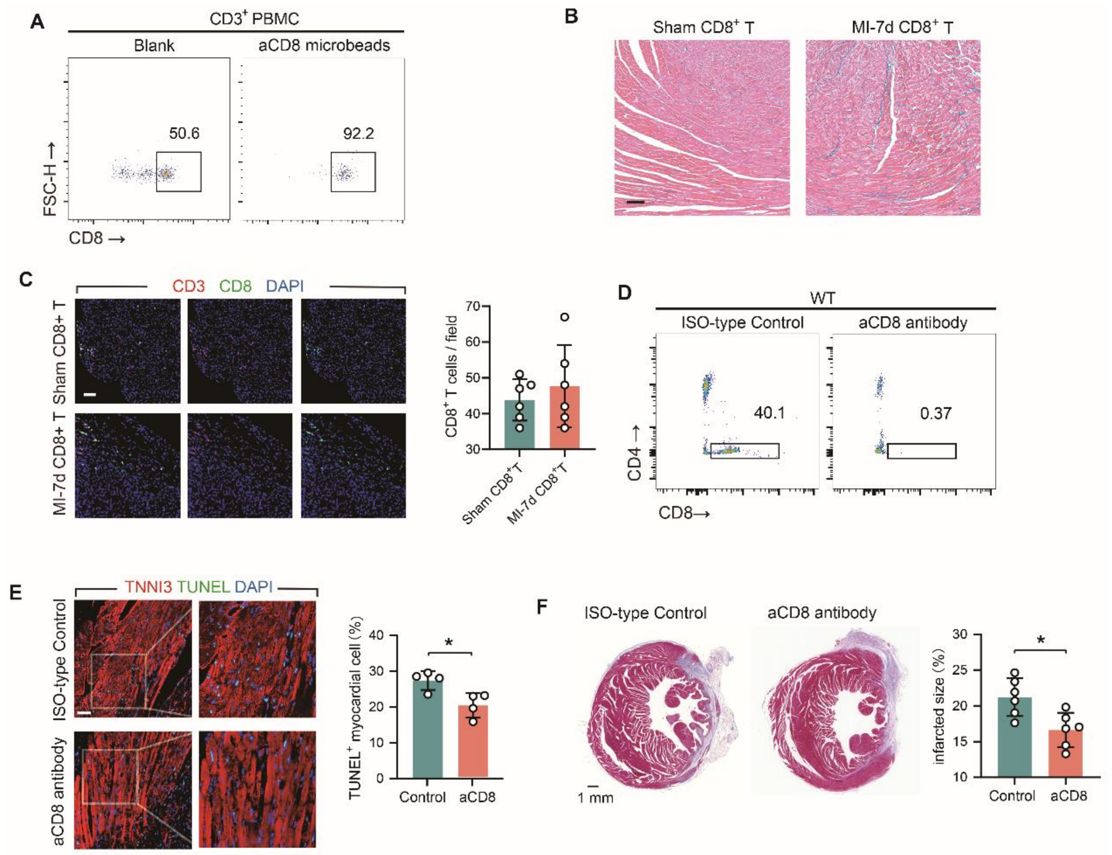

Figure S2 Adoptive transfer of MI-7d CD8$^+$ T cells results in myocardial inflammatory manifestations. A) Flow cytometry results for verifying the purity of CD8$^+$ T cells isolated using anti-CD8(aCD8) microbeads. 7 days after adoptive transfer of sham and MI-7d CD8$^+$ T cells, **B)** Masson staining of the hearts (Scale bar: 50 μm); **C)** Immunofluorescence staining of CD3 and CD8 (n = 4, Scale bar: 25 μm). Under the effect of CD8 antagonistic antibody: **D)** Representative images of the subsets of CD8$^+$ T cells and CD4$^+$ T cells in PBMC of WT mice; **E)** Immunofluorescence staining of TNNI3 (cardiomyocytes) and TUNEL (apoptotic cells) in MI-7d hearts (n = 4, Scale bar: 50 μm); **F)** The Masson staining results of MI-7d hearts (n = 6, Scale bar: 1 mm). Data are presented as the mean ± SD. Unpaired student t test. *$p < 0.05$.

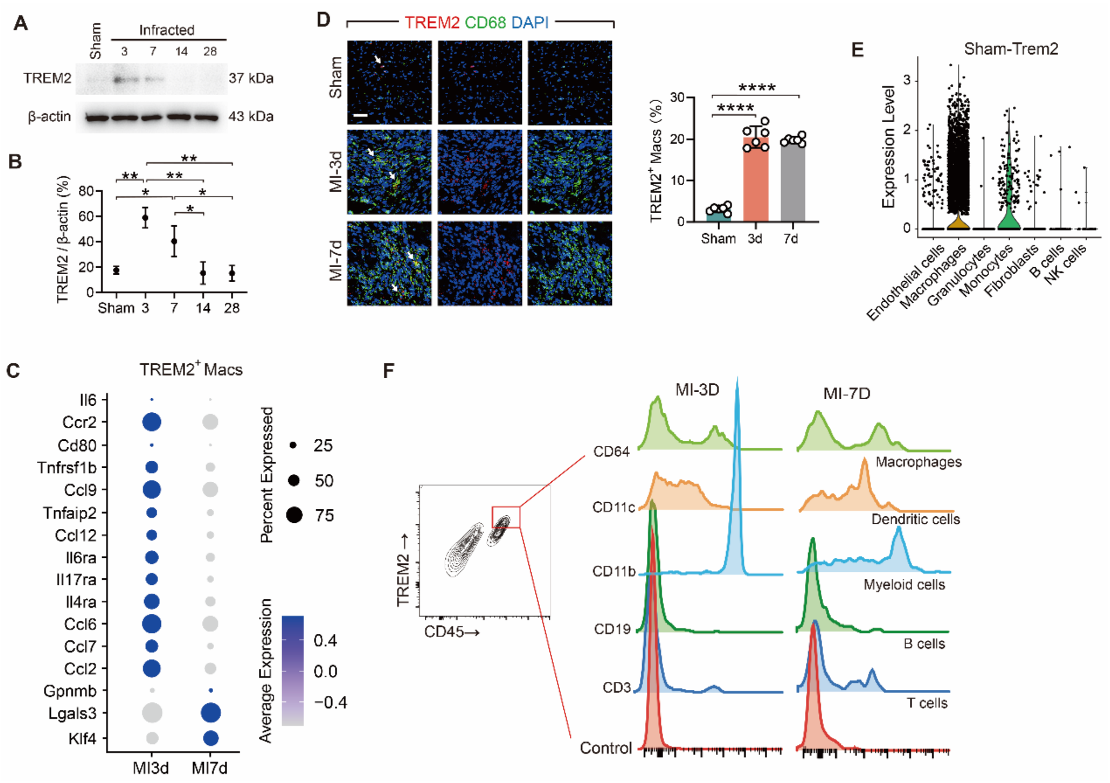

Figure S3 Macrophages exhibit high expression of TREM2 following MI. A) TREM2 protein levels in wild type (WT) mice post-sham surgery, 3, 7, 14, and 28 days after MI. **B)** TREM2 levels were evaluated with β-actin serving as a loading control (n = 3). **C)** Dot plot of representative inflammatory-related genes in TREM2^{+} macrophages (Macs) from single-cell sequencing (scRNA-seq) data at MI-3d and 7d. **D)** TREM2 / CD68 staining in WT hearts after sham surgery or 3 / 7d post-MI (Scale bar: 25 μm) and quantitative analyses of CD68^{+}TREM2^{+} cells (WT n = 6). **E)** Violin plots of TREM2 expression across different cell populations in scRNA-seq data of sham mice. **F)** Expression of TREM2^{+} cell populations in macrophages (CD64), dendritic cells (CD11c), myeloid cells (CD11b), T cells (CD3), B cells (CD19) and isotype controls in the infarcted hearts.

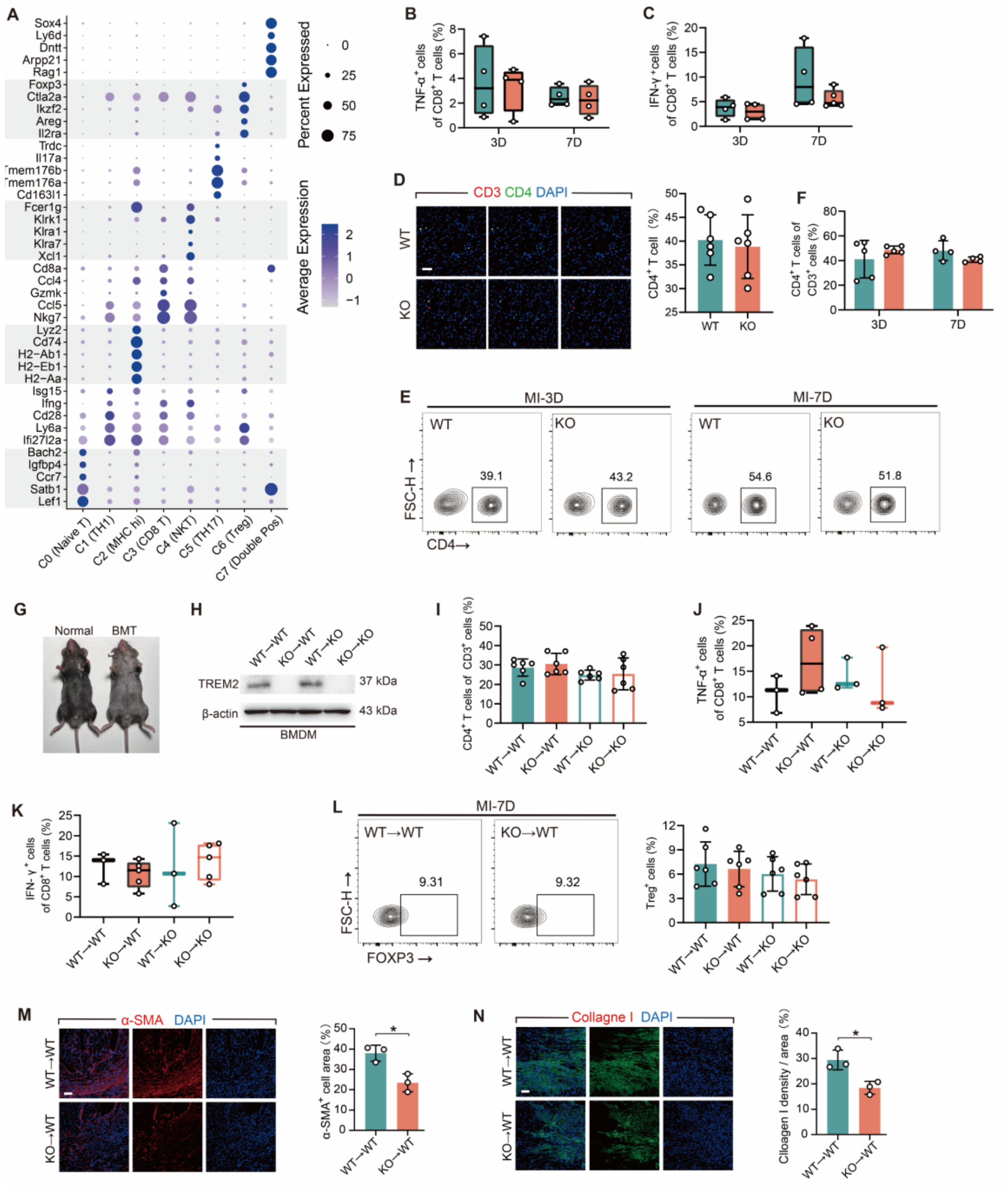

Figure S4 TREM2 does not affect CD4$^+$ T cell infiltration and CD8$^+$ T cell cytotoxicity after myocardial infarction. A) Dot plot of gene expression characteristics of T cell subsets in WT and TREM2 KO MI-7d single-cell sequencing data. Flow cytometry analysis of **B)** IFN-γ$^+$, **C)** TNF-α$^+$ proportions within CD8$^+$ T cells in WT / KO infarcted hearts at MI-3 / 7d (n = 4). **D)** Immunofluorescence images & quantification: CD3 / CD4 staining in infarct hearts, WT / KO, MI-7d (n = 6, Scale

bar = 25 μm). **E, F)** Flow cytometry analysis of $CD4^+$ T cells in WT / KO infarct hearts at MI-3 / 7d. **G)** Images of mice with white fur at 8 weeks post-bone marrow transplantation (BMT). **H)** Western blot results for TREM2 protein in bone marrow cells from various groups at 8 weeks post-BMT, indicating successful bone marrow reconstruction. Flow cytometry analysis of **I)** $CD4^+$ T cells, **J)** IFN-γ^+ , **K)** TNF-α^+ proportions within $CD8^+$ T cells (n = 3, 5); **L)** Treg cells ($CD4^+FOXP3^+$) in BMT infarcted hearts at MI-7d (n = 6). **M)** α-SMA and **N)** Collagen I of WT→WT and KO→WT mice at 7 days post-MI (Scale bar: 25 μm, n = 3). WT→WT: Bone marrow cells from WT mice were transplanted into WT mice. WT→KO: Bone marrow cells from WT mice were transplanted into KO mice. KO→WT: Bone marrow cells from KO mice were transplanted into WT mice. KO→KO: Bone marrow cells from KO mice were transplanted into KO mice. Data are presented as the mean ± SD. Unpaired student t test or one-way ANOVA, Tukey test.

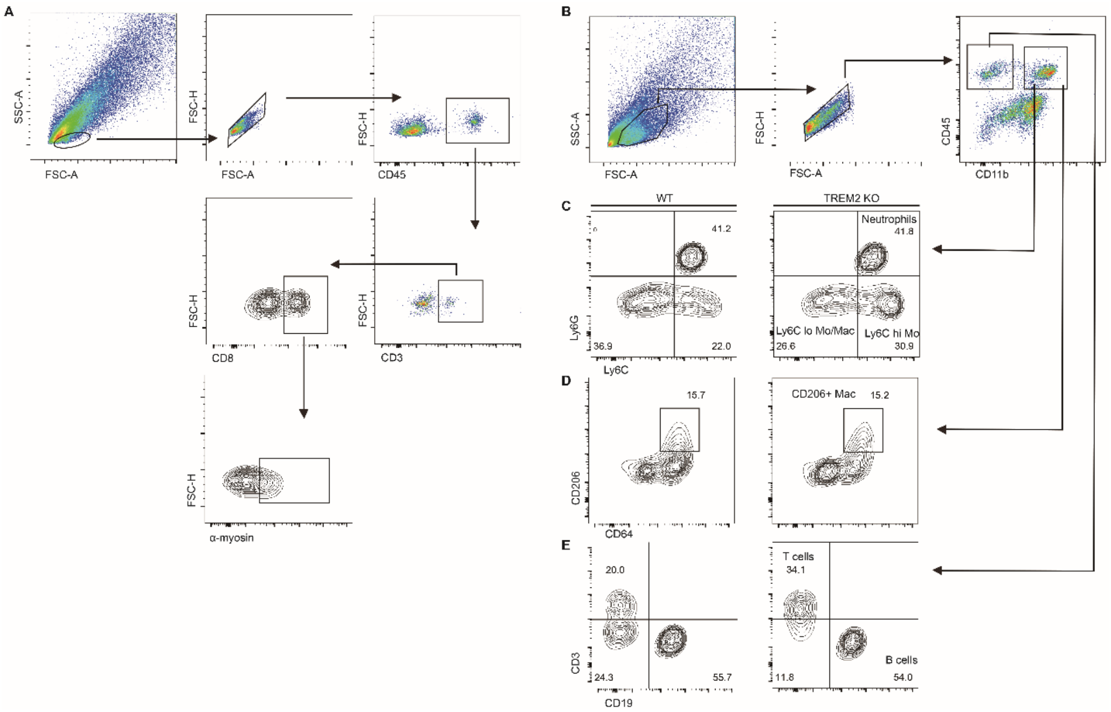

Figure S5 Flow Cytometry Gating Strategy. Gating strategy for flow cytometry analyses of: **A)** α-myosin peptide$^+$ CD8$^+$ T cells. **B)** CD45$^+$CD11b$^+$ and CD45$^+$CD11b$^-$ cells; **C)** neutrophils, Ly6C^{hi} monocytes (Mo), and Ly6C^{lo} monocyte-derived macrophages (Mo/Mac); **D)** CD206$^+$ macrophages; **E)** T cells and B cells.

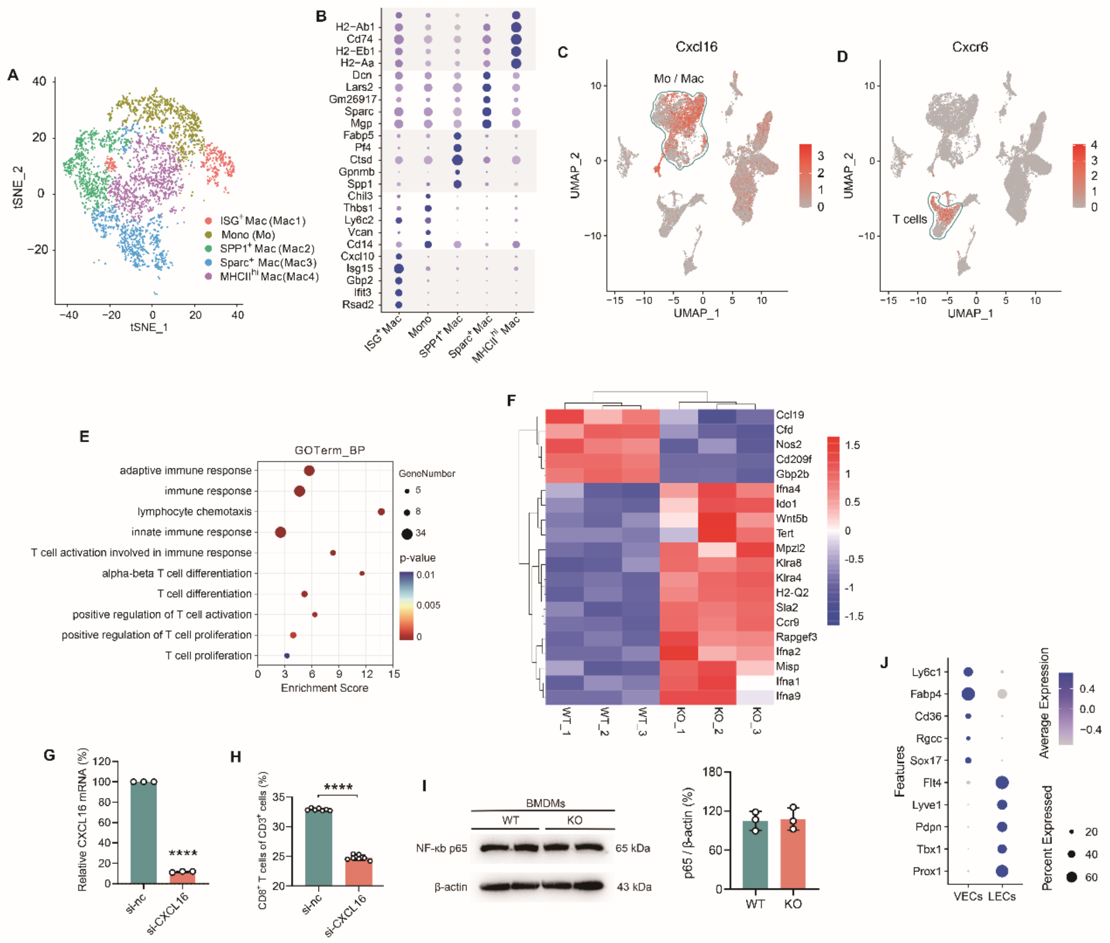

Figure S6 TREM2 Deficiency promotes CD8$^+$ T cell infiltration after MI. A) UMAP distribution of monocyte-derived macrophage cell subsets in MI-7D single-cell RNA sequencing data from WT and TREM2 KO mice and **B)** dot plot of gene expression characteristics. **C, D)** Expression of Cxcl16 and Cxcr6 in monocyte-derived macrophage (Mo/Mac) and T cell clusters (indicated by green circles). **E)** GO (BP) analysis of differentially expressed genes in bulk RNA-seq of macrophages (CD45$^+$CD11b$^+$CD64$^+$) in the infarcted hearts of WT and TREM2 KO mice at MI-7d. **F)** Heatmap showing the top 20 differentially expressed genes. **G)** qPCR results of bone marrow - derived macrophages (BMDMs) from TREM2 knockout (KO) mice transfected with control siRNA (si - nc) and CXCL16 siRNA (si - CXCL16). **H)** The proportion of migrated CD8$^+$ T cells in BMDMs culture medium treated with si-nc and

si-CXCL16 (n = 5). **I)** NF-κb p65 protein levels in BMDMs and the quantitative analysis. Unpaired student t test. **J)** Dot plot depicting the marker genes that determine the classification criteria for vascular endothelial cells (VECs) and lymphatic endothelial cells (LECs).

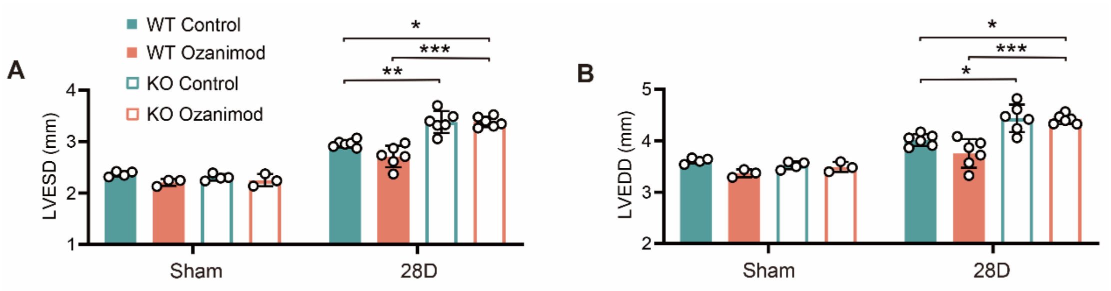

Figure S7 TREM2 deficiency deteriorates cardiac function. A) Echocardiographic analysis of left ventricular end-systolic diameter (LVESD) and **B)** LV end-diastolic diameter (LVEDD) of WT and TREM2 KO mice (n = 4, 6). Data are presented as the mean ± SD. Unpaired student t test. *$p < 0.05$, **$p < 0.01$, ***$p < 0.001$

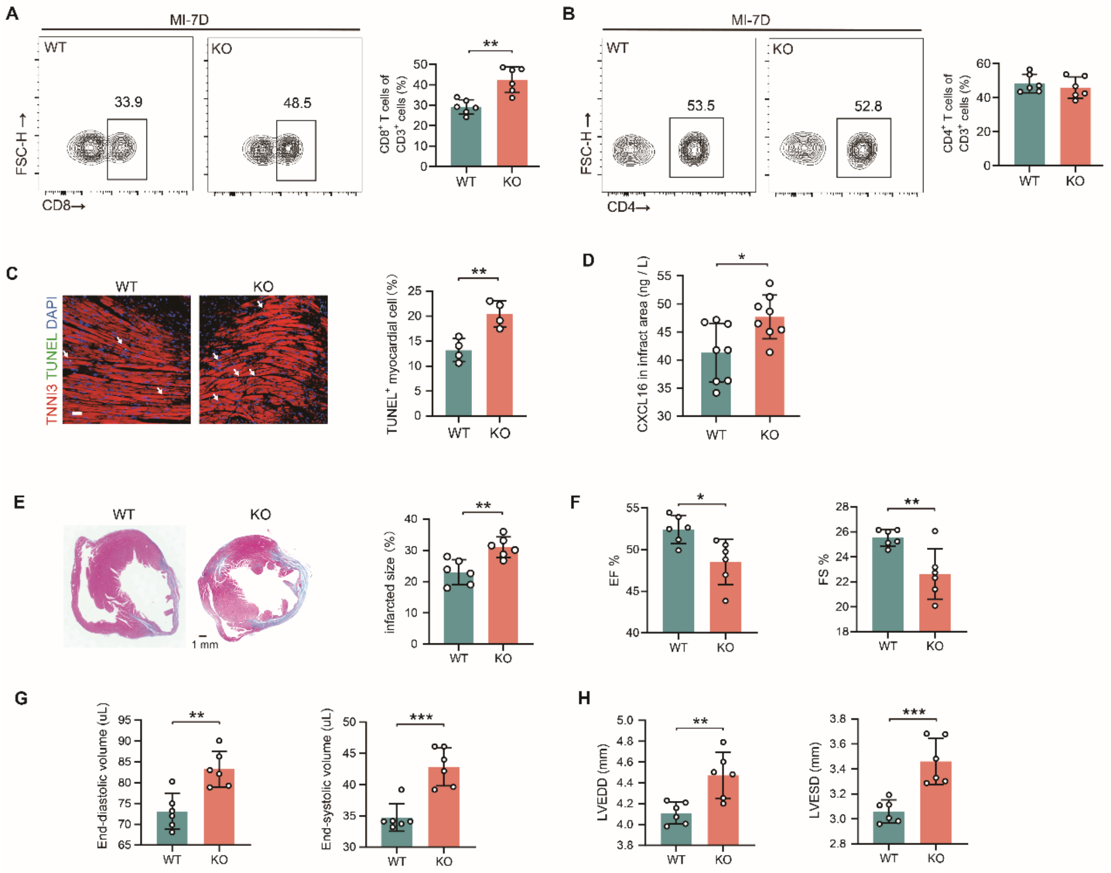

Figure S8 TREM2 deficiency exacerbates CD8$^+$ T cell infiltration in the infarcted area of female mice. Flow cytometry analysis of **A)** CD8$^+$ T cells; **B)** CD4$^+$ T cells in WT / KO infarcted hearts of female mice at MI-7d (n = 6).**C)** Immunofluorescence images and quantification of TUNEL$^+$ myocardial cells in infarct border zone of female mice, MI-7d (Scale bar: 50 μm, n = 4). **D)** ELISA results of CXCL16, infarct areas of female mice, MI-7d (n = 8). **E)** Masson staining on MI-28d and scar area quantification (n = 6, Scale bar: 1 mm). Echocardiographic analysis of **F)** LV ejection fraction (EF), LV fractional shortening (FS), **G)** LV end-diastolic volume, end-systolic volume (n = 6), **H)** LV end-systolic diameter (LVESD) and LV end-diastolic diameter (LVEDD) of WT and TREM2 KO female mice. Data are presented as the mean ± SD. Unpaired student t test. *$p < 0.05$, **$p < 0.01$.

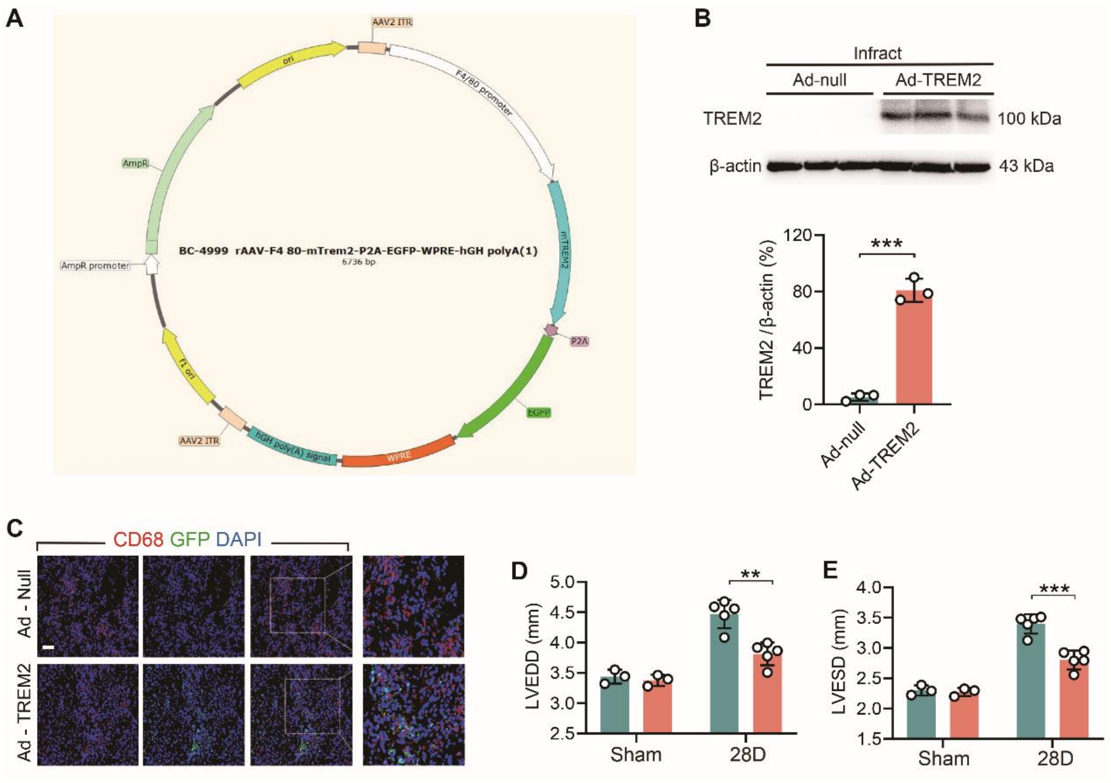

Figure S9 Overexpression of TREM2 improves cardiac function after MI. A) Schematics of adenoviral vector expressed mouse TREM2 driven by F4/80 promoter. **B)** TREM2 protein levels 3 weeks post-injection of TREM2 adenovirus (Ad-TREM2) or control vector (Ad-null) (n = 4). **C)** Immunofluorescence staining confirming TREM2 transfection 7 days post-injection of adenovirus (Scale bar: 25 μm). Echocardiographic analysis of **D)** left ventricular end-diastolic diameter (LVEDD) and **E)** LV end-systolic diameter (LVESD) (n = 3, 5). Data are presented as the mean ± SD. Unpaired student t test. $**p < 0.01$, $***p < 0.001$.

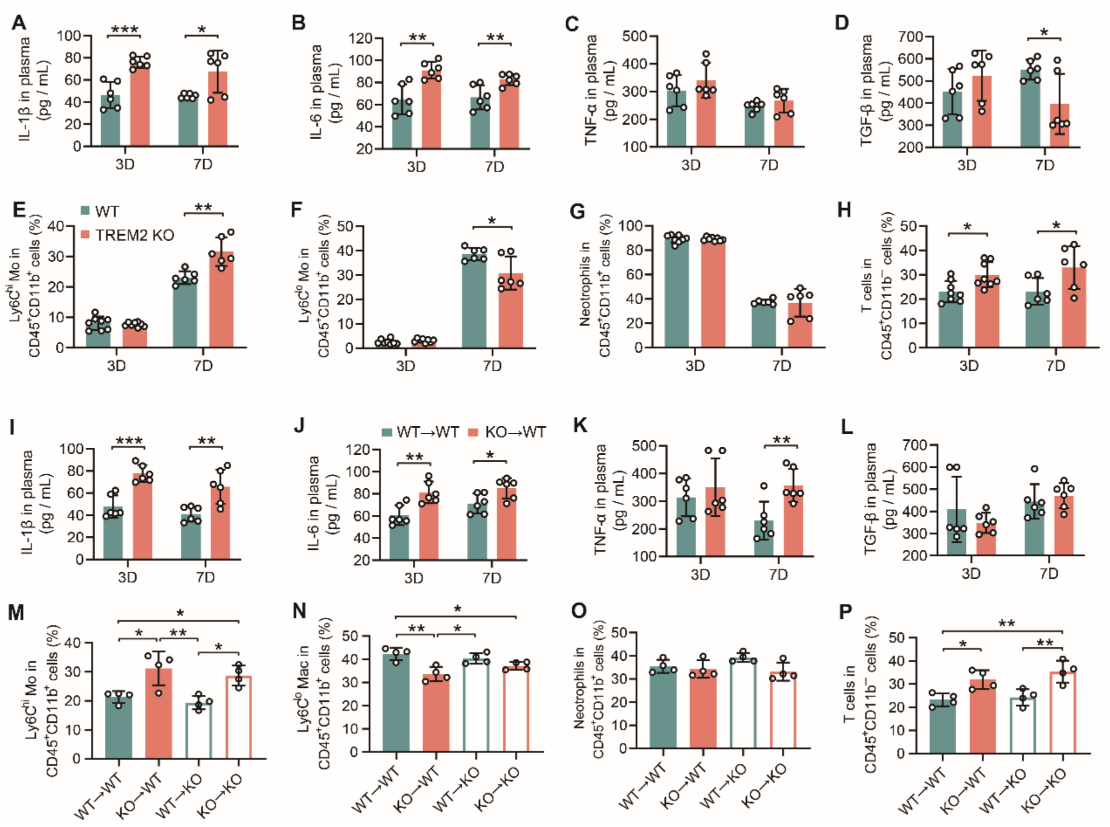

Figure S10 TREM2 deficiency delays the resolution of inflammation after MI. ELISA Outcomes. **A**) IL-1β; **B**) IL-6; **C**) TNF-α; and **D**) TGF-β in the peripheral blood of WT and KO mice, 3/7 days post-MI (n= 6). Flow cytometry analysis of the proportions of **E**) Ly6C^{hi} monocytes (Mo), **F**) Ly6C^{lo} macrophages (Mac), **G**) neutrophils, and **H**) T cells in the infarcted hearts (n = 8, 6). ELISA Results: **I**) IL-1β, **J**) IL-6, **K**) TNF-α, **L**) TGF-β in Peripheral Blood of WT→WT & KO→WT Mice, 3/7 days post-MI (n = 6). Flow cytometry analysis of the proportions of: **M)** Ly6C^{hi} Mo, **N)** Ly6C^{lo} Macs, **O)** neutrophils, **P)** T Cells in infarcted and border zones, 4 groups of bone marrow transplantation mice, MI-7d (n = 4). WT→WT: Bone marrow cells from WT mice were transplanted into WT mice. WT→KO: Bone marrow cells from WT mice were transplanted into KO mice. KO→WT: Bone marrow cells from KO mice were transplanted into WT mice. KO→KO: Bone marrow cells from KO mice were

transplanted into KO mice. Data are presented as the mean ± SD. Student unpaired t tests or one-way ANOVA, Dunnett's test. *$p < 0.05$, **$p < 0.01$.

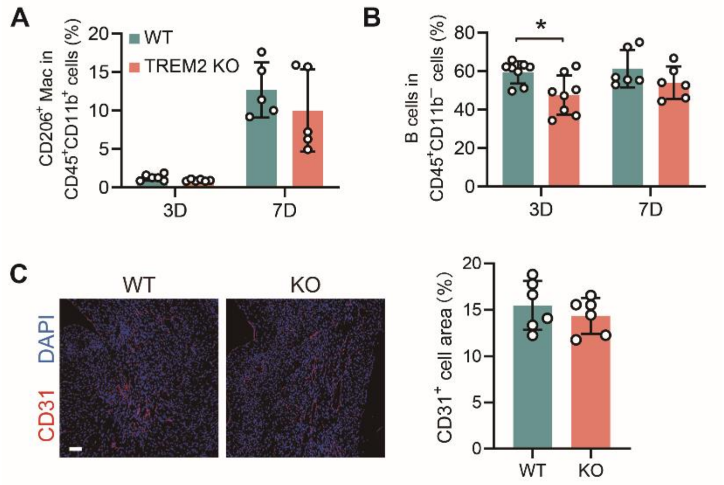

Figure S11 TREM2 does not affect the proportion of CD206$^+$ macrophages and angiogenesis. A, Flow cytometry analysis showing the proportions of CD206$^+$ macrophages (n = 6). **B**, B Cells in infarcted and border zones: WT vs. TREM2 KO, MI-3d & MI-7d (n=8, 6). **C**, Immunofluorescence images and quantification of CD31 expression in infarct hearts, MI-7d (n=6, Scale bar=25 μm). Unpaired student t test.
